# Bayesian integration of genetics and epigenetics detects causal regulatory SNPs underlying expression variability

**DOI:** 10.1038/ncomms9555

**Published:** 2015-10-12

**Authors:** Avinash Das, Michael Morley, Christine S. Moravec, W. H. W. Tang, Hakon Hakonarson, MAGNet Consortium, Euan A. Ashley, Jeffrey Brandimarto, Ray Hu, Mingyao Li, Hongzhe Li, Yichuan Liu, Liming Qu, Pablo Sanchez, Kenneth B. Margulies, Thomas P. Cappola, Shane Jensen, Sridhar Hannenhalli

**Affiliations:** 1 Center for Bioinformatics and Computational Biology, University of Maryland, College Park, Maryland 20742, USA; 2 Perelman School of Medicine, University of Pennsylvania, Philadelphia, Pennsylvania 19104-5159, USA; 3 Department of Cardiovascular Medicine, Heart and Vascular Institute, Cleveland Clinic, Cleveland, Ohio 44195, USA; 4 The Childrens Hospital of Philadelphia, Philadelphia, Pennsylvania 19104-5159, USA; 5 The Wharton School, University of Pennsylvania, Philadelphia, Pennsylvania 19104-6340, USA; 6 Stanford Center for Inherited Cardiovascular Disease, Stanford University School of Medicine, Stanford, CA 94305, USA; 7 Penn Cardaiovascular Institute and Department of Medicine, Perelman School of Medicine, University of Pennsylvania, Philadelphia, PA 19104, USA; 8 Department of Biostatistics and Epidemiology, University of Pennsylvania Perelman School of Medicine, Philadelphia, PA 19104, USA

## Abstract

The standard expression quantitative trait loci (eQTL) detects polymorphisms associated with gene expression without revealing causality. We introduce a coupled Bayesian regression approach—eQTeL, which leverages epigenetic data to estimate regulatory and gene interaction potential, and identifies combination of regulatory single-nucleotide polymorphisms (SNPs) that explain the gene expression variance. On human heart data, eQTeL not only explains a significantly greater proportion of expression variance but also predicts gene expression more accurately than other methods. Based on realistic simulated data, we demonstrate that eQTeL accurately detects causal regulatory SNPs, including those with small effect sizes. Using various functional data, we show that SNPs detected by eQTeL are enriched for allele-specific protein binding and histone modifications, which potentially disrupt binding of core cardiac transcription factors and are spatially proximal to their target. eQTeL SNPs capture a substantial proportion of genetic determinants of expression variance and we estimate that 58% of these SNPs are putatively causal.

## Abstract (web summary)

Das *et al*. present a novel Bayesian approach called expression Quantitative Trait enhancer Loci (eQTeL), which effectively integrates genetic and epigenetic information to identify combination of regulatory genomic variants underlying expression variance. Using various functional data, the authors show the variants identified by eQTeL are likely to be causal.

## Graphical abstract

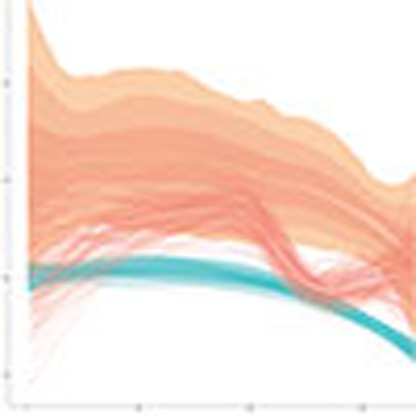

Numerous expression quantitative trait loci (eQTL) studies have been performed to determine the cell-type-specific regulatory architecture of the human genome^1^. However, since single-nucleotide polymorphisms (SNP) within a linkage disequilibrium (LD) region are statistically indistinguishable from each other, these studies essentially reveal LD blocks that are associated with a gene expression but do not reveal the potential causative regulatory SNPs, which limits the utility of these studies^2^,^3^,^4^,^5^,^6^. The recent explosion of epigenetic data has made it possible to detect cell-type-specific regulatory regions^5^,^6^,^7^,^8^,^9^, which can be used to distinguish regulatory SNPs from non-regulatory SNPs in LD blocks.

Recently, a few approaches have incorporated regulation specific epigenetic data into association studies^5^,^6^,^7^,^8^,^9^,^10^. However, to prioritize eQTL SNPs, these methods have utilized the regulatory information either retrospectively or to estimate an empirical prior probability for the SNPs. Such approaches are prone to missing regulatory SNPs with small effects due to the severe multiple testing correction (or sparsity constraints)^1^. Furthermore, these approaches ignore interaction between the region harbouring the SNP and the target gene, which is useful in identifying regulators specific to a gene. Multiple SNPs are known to regulate single genes^11^, yet many current methods^8^,^9^,^11^ limit the number of causal SNPs per gene to a single SNP. In this paper, we introduce a new method, expression quantitative trait enhancer loci (eQTeL), which addresses these limitations. It identifies combination of regulatory SNPs—including SNPs with small effect sizes—that jointly determine expression variance.

eQTeL is a fully Bayesian approach (Fig. 1), which infers cis regulatory polymorphisms underlying gene expression variability by integrating: (i) genotype and gene-expression variance across individuals; (ii) epigenetic data in appropriate cell types^10^,^12^; (iii) DNAse I hypersensitivity (DHS) variance of SNPs and promoters across cell types^13^; (iv) expression variance of genes across multiple cell types; (v) LD blocks^14^; and (vi) imputed haplotypes inferred from the 1,000 Genomes Project^15^. Our approach addresses a number of key methodological challenges. First, it systematically integrates three characteristics of a causal regulatory eQTL, that is, correlation with the target genes expression across individuals, the regulatory properties of the harbouring region, and interaction with the target gene. Second, it can account for heterogeneity of regulatory regions in terms of different combinations of epigenetic marks. Third, to learn the regulatory model, eQTeL leverages regulatory polymorphisms that are not associated with gene expression in addition to expression-regulators. Fourth, it interrogates the LD structure to find the optimal combination of explanatory SNPs. Fifth, it implements a hierarchical scheme to select a sparse set of SNPs, while simultaneously explaining a maximal fraction of gene expression variance. Finally, eQTeL is scalable to large datasets.

We statistically validated our method using human heart data as well as realistic simulated data and demonstrated that it can predict an individual's expression from the genotype more accurately compared to other methods. SNPs identified by our method include regulatory SNPs with small effect sizes. Further assessment of functional relevance of identified SNPs suggest that they tend to (i) overlap a high resolution DNAse footprint, (ii) have an allele-specific DNAse footprint, (iii) preferentially disrupt putative binding of core cardiac regulators and (iv) be spatially proximal to their putative target gene. We also estimate that 58% of SNPs identified by eQTeL (which we call eeSNPs, Supplementary Data 1) are likely to be causal. Collectively, these results strongly suggest that eeSNPs have functional role.

## Results

### Quantitative Trait enhancer Loci (eQTeL) model

**Figure 1:**
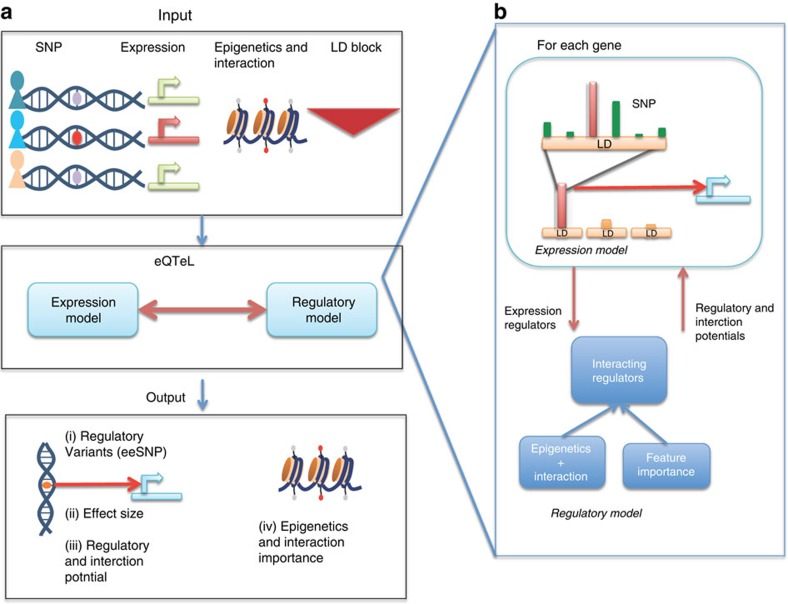
Overview of eQTeL model. (**a**) Input and output of eQTeL. eQTeL takes genotype and gene expression across samples, epigenetic and interaction features for each SNP and LD block as input. It outputs regulatory SNPs and their target genes, their effect sizes and regulatory-interaction potentials, as well as estimated feature importance of each epigenetic and interaction feature. (**b**) eQTeL is composed of two coupled regression models (i) a Bayesian variable selection with informative priors models expression as a linear combination of SNPs. Given the regulatory and interaction priors, this hierarchical model first identifies LD blocks and then combinations of SNPs that explains expression variance and that also have high regulatory and interaction potentials. (ii) A Bayesian logistic regression specifies the regulatory and interaction potential as linear model of epigenetic and interaction features in semi-supervised manner. The logistic regression passes the regulatory and interaction potentials to the variable selection model, while the variable selection model passes expression-regulators to the logistic regression model.

We first provide a broad overview of the eQTeL model and further details can be found in Methods. As illustrated in Fig. 1, eQTeL is composed of two Bayesian regression models, an expression model and a regulatory model, which are coupled through message passing. The expression model is a Bayesian variable selection model^16^,^17^ which explains the gene expression variance among samples as a linear function of SNP alleles. A distinct feature of the expression model is that it uses informative prior for each SNP, which depends on the SNPs regulatory^6^ and interaction potential. The regulatory model, which is common for all genes, uses a Bayesian logistic regression^18^ to estimate that informative prior as a probabilistic function of epigenetic and interaction features. Known expression regulators can be used to train the regulatory model, while an accurate model of regulatory and interaction potential can help to identify expression regulators. The expression model then passes current estimates of expression regulators to the regulatory model, which in passes current estimates of regulatory and interaction priors for each SNP back to the expression model. eQTeL starts with estimating expression regulators assuming equal priors for each SNP and then, using current estimates of expression-regulators, trains the regulatory-model. In turn, current estimates of regulatory and interaction potential are used as informative priors to re-estimate expression regulators. This iterative process continues until convergence. Thus, our eQTeL model gradually improves estimation accuracy by joint learning.

In our approach (see equations below and Methods for details), expression *Y* relates to candidate SNPs *X* via a standard normal linear model^16^,^19^,^20^ with noise *σ*^2^. However, for each SNP *β*, its effect size is non-zero only if its regulatory-interaction indicator *γ* is 1, which depends on a function *ϕ*′*(θ)* of regulatory-interaction potential *θ* (Methods). The potential *θ* of a SNP is modelled as a combination of (i) features for regulatory potential and (ii) features for SNP-gene interaction P, via a logistic function. Vector *α* represents feature weights that are shared across all genes, thus we learn a single genome-wide model of regulators. This choice of modelling *α* obviates the need to explicitly scale genetic and epigenetic factors.


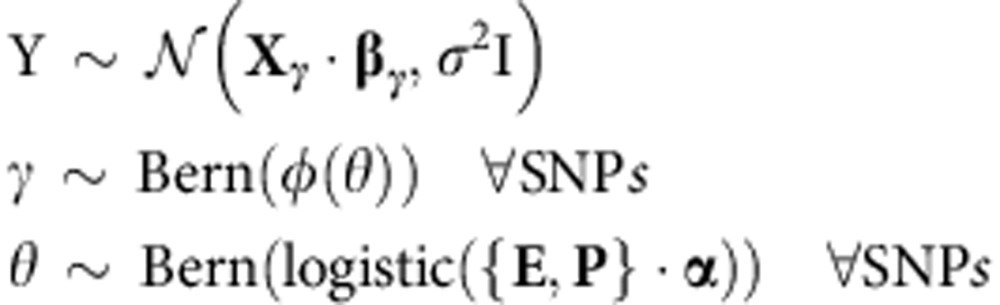


We use Markov chain Monte Carlo^21^ to infer all model parameters jointly (Supplementary Note 1). At each iteration of the sampler, the decision whether a region is a regulator (that is, *θ*=1) depends not only on correlation between corresponding SNP and gene but also on the regulatory and interaction features, as well as the current estimates of feature weights. This leads to a semi-supervised^22^,^23^ clustering of SNPs into regulators and non-regulators (Supplementary Note 1). Our Markov chain Monte Carlo implementation explicitly uses LD^24^ block information to judiciously choose combination of regulatory SNPs by sampling over the model space hierarchically^21^ at the top level it explores combinations of LD blocks and at the lower level it explores the sparse set of SNPs within each LD block that optimally explain the expression-variance (Fig. 1, Methods, Supplementary Note 1, Supplementary Fig. 1). This approach results in a superior exploration of the model space relative to approaches that disregard the LD structure. eQTeL uses a Rao–Blackwell estimate of *θ* that improves the mixing rate (Supplementary Fig. 1) of the sampler and leads to robust competition between SNPs within a LD block (Fig. 1). Further, the overall sparsity constraint (equivalent to a multiple testing correction in non-Bayesian approaches) of eQTeL is controlled by two factors: (i) the fraction of SNPs that are interacting-regulators and (ii) the fraction of interacting-regulators that are expression-regulators. This allows for a less conservative sparsity constraint and makes it possible to identify SNPs with small effect sizes which are typically missed by alternative approaches because of severe multiple testing correction. eQTeL assumes Normal priors on *α*. Finally, eQTeL implementation allows an option to select a subset of epigenetic factors important for estimating regulatory potential through Bayesian variable selection model.

### eQTeL detects expression regulatory SNP in MAGNet

We applied eQTeL to genotype and gene expression data for 313 human hearts (procured by MAGNet consortium (www.med.upenn.edu/magnet/)) and compared with the performance of other eQTL methods (Supplementary Notes 2 and 3). To determine regulatory and interaction potentials, we used 95 epigenetic and interaction features (Supplementary Fig. 2) for primary tissues and cell lines of heart from ENCODE and Roadmap Epigenome project^10^,^12^. For expediency we selected 1,880 genes with expression deemed to have a significant genetic component according to the univariate eQTL^11^,^25^.

**Figure 2:**
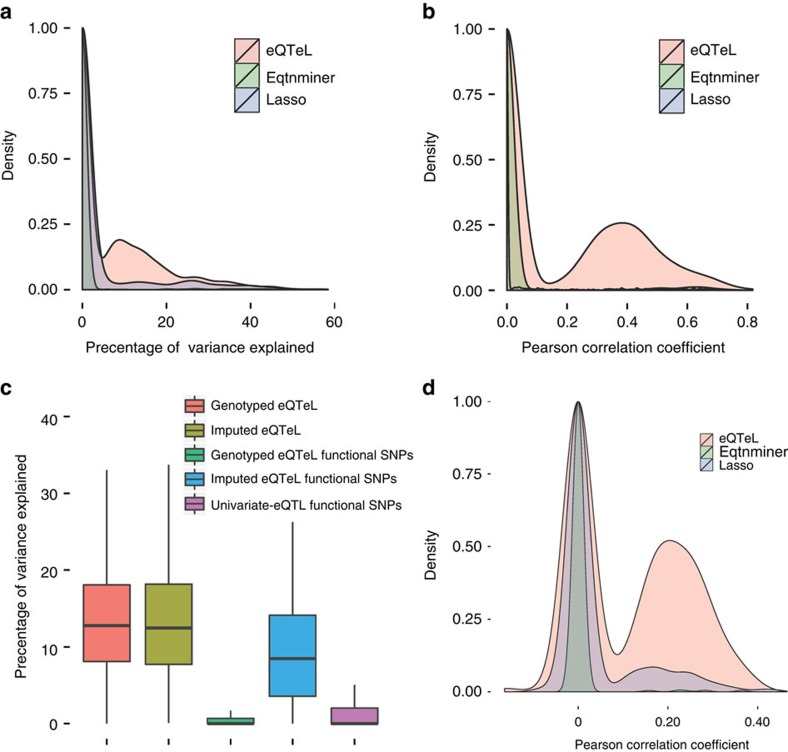
Comparative performance of different methods applied to human heart data (MAGNet). The analysis is based on 2428 SNPs identified by eQTeL for which posterior probability of selection >0.5. To ensure the same total number of SNPs selected by eQTeL, eqtnminer and LASSO: for eqtnminer we sort SNPs based on posterior probability and for LASSO based on absolute estimated effect size and then selected top 2,428 SNPs. (**a**) Explained expression variance based on three representative methods on human heart data. (**b**) Accuracy of predicted expression of three methods. (**c**) Explained expression variance for human heart data by potentially functional (approximated by overlap with a footprint) genotyped SNPs and imputed SNPs. (**d**) Cross-data set generalization of MAGNet eeSNPs: expression predictability in GTEx by eeSNPs identified in MAGNet.

Consistent with its ability to explain a greater expression variance, eQTeL also predicts expression of genes much more accurately compared with other methods (Fig. 2b). The mean (cross-validated) Pearson correlation coefficient between predicted and actual expression is 0.176±0.065 (in contrast with 0.025 for eqtnminer^8^ and 0.088 for LASSO^26^). The bimodality of distribution of correlation coefficient implies that for a subset of genes, the expressions are highly predictable by eQTeL.

Because of its ability to discriminate among multiple SNPs based on regulatory and interaction potentials, eQTeL is expected to be much more advantageous on imputed data, which has a substantially greater number of linked SNPs. To confirm this, we imputed^27^ ∼6.5 million SNPs using the 1,000 Genome Project data^15^. Note that each imputed SNP is derived from the reference SNPs using the linkage information, and cannot be any more associated (in a statistical sense) with the gene expression than the reference SNPs, and therefore are not expected to increase the explained variance (as evident from Fig. 2c). However, eQTeL with imputation is expected to improve detection of causal functional SNPs compared with the genotyped SNPs^10^,^11^. Therefore, restricting our search to potentially functional SNPs, imputed SNPs should explain the expression better. Restricting our analysis only to SNPs mapped to a DNAse footprint (as a proxy for putative functional SNPs), the relative advantage of imputation with eQTeL becomes evident (Fig. 2c). Indeed, with imputed data, there is no significant improvement in detection of likely causal SNPs if standard eQTL approaches are used. Therefore it becomes imperative to use an integrative approach, such as eQTeL, in the presence of a large number of linked SNPs (Fig. 2c).

To validate eeSNPs in an independent cohort, we analysed expression and genotype of 85 left ventricle samples from GTEx^1^ (Supplementary Note 2). We note that compared to an exhaustive eQTL, eQTeL cannot identify novel associated loci, but instead is designed to identify putatively causal SNPs within an associated locus. We found that 18.9% of eGenes detected in MAGNet replicates in GTEx (Supplementary Data 2). To assess the relative generalizablity of eQTeL in independent cohort, using the eeSNPs identified by eQTeL in MAGNet, we estimated the explained variance in GTEx. We repeated this for other methods while controlling for the number of eeSNPs as well as other regularization procedures. While, as expected due to the differences in the datasets, the cross-cohort explained variance is lower than that within MAGNet (Fig. 2b versus 2d), relative to other methods, eQTeL exhibits substantially and significantly greater (in both cases Wilcoxon test *P* value between eQTeL and other methods is <1.0 × 10^−16^) cross-data set generalizability (Fig. 2d, Supplementary Fig. 3).

### eQTeL detects causal SNPs in semi-synthetic data

**Figure 3:**
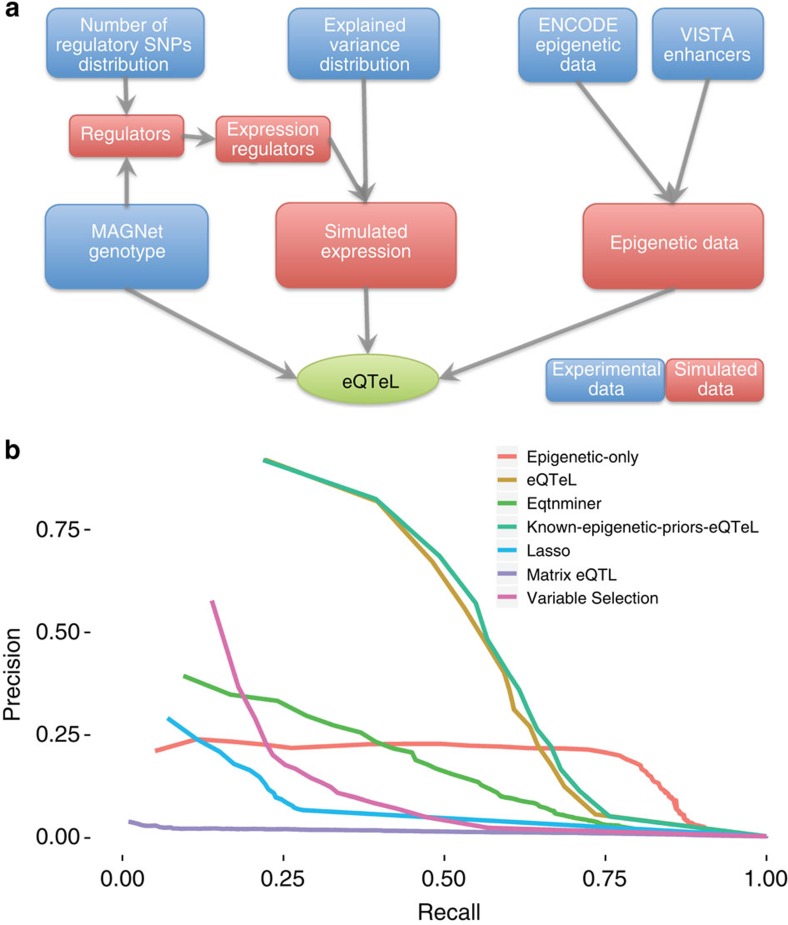
eQTeL identify causal SNP accurately in semi-simulated data. (**a**) Design of simulaton study: simulation study uses (i) 174800 SNPs from MAGNet Genotype (874 SNPs per gene) data for 313 samples, (ii) distribution of number of expression-regulators per gene from MAGNet data, (iii) distribution of explained expression variance estimated from MAGNet data, (iv) ENCODE epigenetic data for heart cell lines and (v) distribution of epigenetic data for regulators VISTA heart enhancers. Expression regulators per gene were chosen amongst regulators (1% of MAGNet SNPs). Using allele status of expression regulators in 313 samples expression of 200 genes was generated such that explained variance distribution matches MAGNets explained variance. Epigenetic data for regulators were generated using the epigenetic distribution estimated from VISTA heart enhancers. (**b**) Comparative performance assessment on simulated data. Methods include (i) Matrix-eQTL^11^,^25^ (univariate-eQTL): univariate regression, (ii) LASSO^44^: L1 regularizer multivariate regression, (iii) variable selection^17^: Bayesian variable selection, (iv) eqtnminer^8^: Bayesian variable selection with empirical-priors, (v) epigenetic-only: epigenetic feature weights derived from verified enhancers and used to prioritize SNPs, (vi) eQTeL: proposed method and (vii) known-epigenetic-priors-eQTeL: eQTeL with fixed epigenetic priors as in epigenetic-only. Number of SNPs each methods were controlled.

To demonstrate that eQTeL can accurately identify putatively causal SNPs, we use a synthetic data evaluation (Fig. 3a) (for additional details refer to Methods). We used 174,800 SNP probes along with their genotypes from 313 MAGNet samples that were within 1 MB from transcription start of 200 genes (Methods). Since regulatory region may have no effect on genes included in our analyses and yet can contribute to learning the regulatory-model, eQTeL makes a distinction between a regulator and a gene-specific expression-regulator. This distinction was made explicitly in our simulation by designating 1% of all SNPs as regulators (as an approximation of previous estimation in humans^28^). We then used a frequency distribution of expression regulators per gene inferred from MAGNet data to randomly choose gene-specific expression-regulators for 200 genes. Using allele status of 313 samples for expression-regulators, we generated gene expression and added random noise such that expected explained variance from simulated data matched MAGNets explained variance (Fig. 2a). We generated the epigenetic features for each SNP using ENCODE epigenetic data and validated heart-enhancers from VISTA^6^. Thus our simulated data closely parallels the experimental data.

Next we applied eQTeL to the simulated data. The precision-recall plot (Fig. 3b) shows that eQTeL significantly outperforms other methods. In fact, the performance of full-eQTeL is close to the theoretically best eQTeL model that uses the original feature weights (see Methods). The previous integrative method eqtnminer^8^,^9^, the only other current method that uses epigenetic data in eQTL, shows only a modest increase in precision compared to methods that do not use epigenetic data.

The immediate effect of increase in precision of detecting expression regulators, especially for SNPs with high regulatory potential, is that eQTeL explains a significantly greater proportion of expression variability (Supplementary Fig. 4). There is also significant improvement in correlation between predicted expression and actual gene expression; mean correlation for eQTeL was 0.298 −+0.02 (compared with 0.18 for eqtnminer and 0.23 for LASSO regression, Supplementary Fig. 5). Note that for this analysis we controlled for the number of SNPs that were selected for each method, using the most explanatory respective SNPs for each method. Overall, eQTeL can accurately identify around 75% of putative causal SNPs (at 40% recall) reinforcing the fact that our method can identify substantial fraction of likely causal genetic determinants of transcriptomic variance.

### eQTeL detects SNPs with small effect sizes

**Figure 4:**
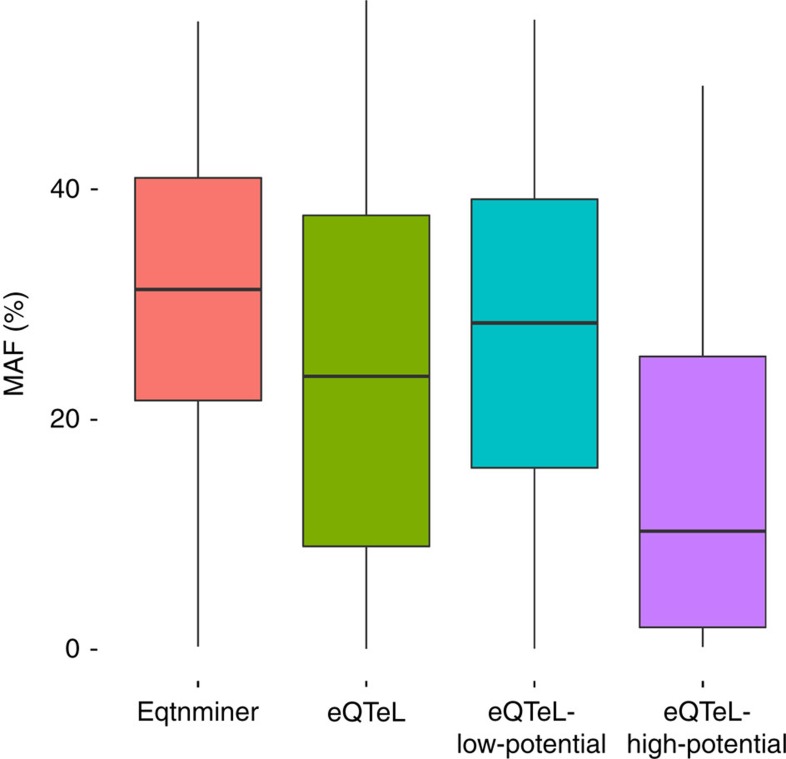
eQTeL increase statistical power to detect small-effect regulatory SNPs: comparsion of effect-size of SNPs detected by eQTeL and eqtnminer. Number of SNPs for each method was controlled. eQTeL can detect SNPs with small effect size if the regulatory potential of SNP is high. eQTeL-high-potential are subset of eeSNPs with interacting-regulatory potential=1 and eQTeL-low-potential are subset with interacting-regulatory potential<0.1.

The statistical power to detect SNPs associated with expression variance (that is, the probability of correctly rejecting the null hypothesis that the SNP is not associated with gene expression) depends on various factors such as sample size, noise to signal ratio, number of hypothesis tested (number of SNPs) and effect size of SNP. The effect size, in turn, depends on the allele frequency of SNP, thus low allele frequency limits statistical power to detect regulatory SNPs^1^,^29^. Another advantage of eQTeL model is that it can detect SNPs with small effect sizes by distributing sparsity between: (a) sparsity in the number of regulators and, (b) sparsity in expression regulators among all regulators. eQTeL employs relatively relaxed sparsity constraints for SNPs that have high regulatory potential and therefore the model has higher statistical power to retrieve a greater fraction of SNPs with low minor allele frequency (small effect sizes) compared to eqtnminer (Fig. 4). Furthermore, eQTeLs statistical power to identify low minor allele frequency SNPs is greater among SNPs with high regulatory-interacting potential (labelled as eQTeL-high in Fig. 4). This trend of differential statistical power is also observed in simulated data, where we know the exact effect size of regulatory SNPs (Supplementary Fig. 6).

eQTeL leverages LD information to judiciously choose combinations of SNPs (per gene) which explains a much greater proportion of expression variance (details in Supplementary Note 2). The power to detect SNPs with low allele frequency is the primary reason that eQTeL captures substantial proportion of causal genetic determinants underlying transcriptomic variance. However, it should be noted that SNPs with small effect sizes are only detected by eQTeL if they have a high regulatory potential.

eQTeLs performance gain is potentially due to two factors: (i) integration of epigenetic data, (ii) allowing multiple causal variants per gene^30^. We assessed relative contribution of the two factors. eQTeLs expression predictability by functional SNPs increases substantially when multiple SNPs per gene were allowed (Supplementary Fig. 7, Supplementary Note 2), supporting a contribution due to multiple explanatory SNPs. However, in the absence of epigenomic data, that is, when using standard LASSO, we do not see a performance gain, and in general, the performance is substantially worse than the performance of eQTeL. This suggests that allowing multiple SNPs per gene is useful specifically when functional information is used.

### eeSNPs lie within protein-bound genomic regions

**Figure 5:**
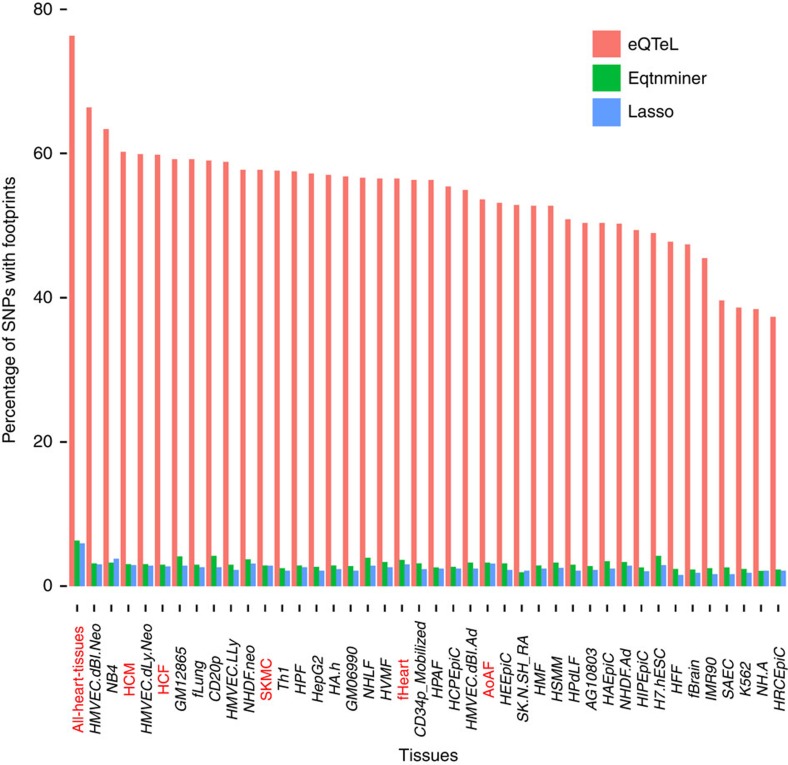
Large fraction of eeSNPs overlaps with DNAse footprint relative to other methods, particularly for heart-related tissues (highlighted in red). This analysis is based on 2,428 SNPs identified by eQTeL for which posterior probability of selection >0.5. For eqtnminer, we selected the best SNP reported for each gene. For LASSO we selected 2,428 SNPs by sorting the effect sizes. We looked at the footprint in 42 cell lines^31^ overlapping the SNP within 25 bps the SNP loci by using bedtools^45^ for each method. The heart-related tissues are highlighted in red in the figure. The left-most bar represents pooled data from all heart-related cell types. Note the relative enrichment of each method remains same even if we control for SNPs per gene in each method.

Putative causal regulatory SNPs are expected to be bound by regulatory proteins. Earlier studies have shown enrichment of regulatory elements near causal SNPs^7^,^8^,^9^,^11^. Since eQTeL and eqtnminer use epigenetic data, which is known to be correlated^10^ with protein binding, we expect to find enrichment of DNAse footprints near the identified regulatory SNPs. Using genome-wide high-resolution DNAse footprint data for 41 cell types^31^, we obtained the fraction of eeSNPs (and control SNPs) overlapping with a footprint; Note that DNAse footprints were not used in eQTeL so they could be used for validation. 76.3% of eeSNP have a footprint overlapping the eeSNP (Fig. 5), in contrast to 6.3% of in SNPs detected by eqtnminer that uses same epigenetic data as eQTeL. The performance of eqtnminer did not improve even if the best SNP per gene were chosen for this analysis. For SNPs chosen by LASSO, which does not use epigenetic data, only 5.95% of SNPs have overlapping DNAse footprints. Only 2% of SNPs identified by Lirnet (for 200 genes) overlap with the DNAse footprints (Supplementary Fig. 8). Using top 8 epigenetic features estimated from eQTeL allowed to improve performance of eqtnminer, but could not bring it up to eeSNPs enrichment level (Supplementary Fig. 9 and Supplementary Note 4). Notably, the DNAse footprint enrichment is high in the four heart-related cell types. This result suggests that majority of SNPs identified by eQTeL coincide with regions of *in vivo* protein binding and are at least 12-fold more likely to be functional than the next closest method.

### eeSNPs exhibit binding and regulatory allele specificity

**Figure 6:**
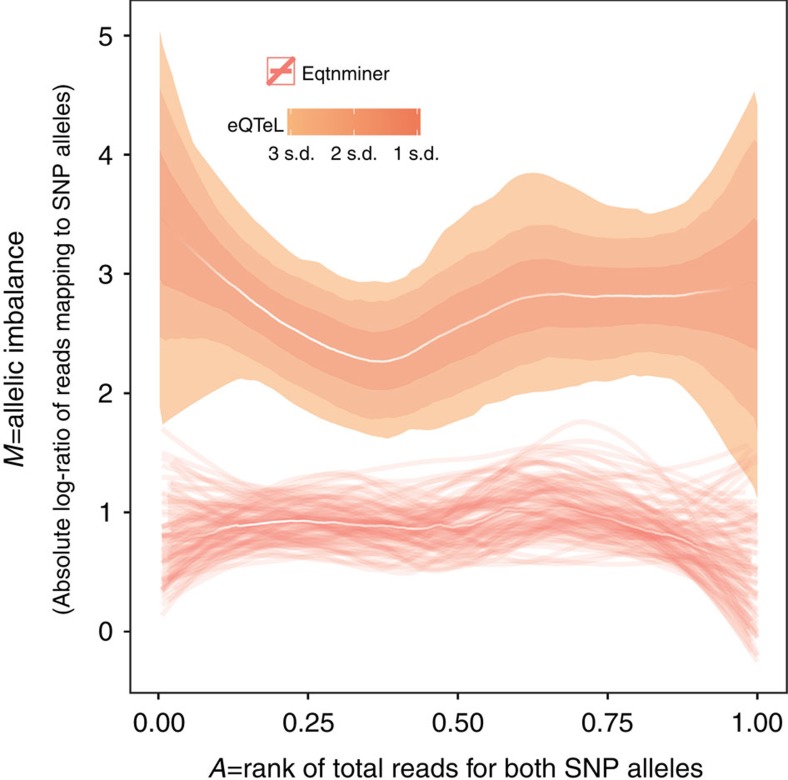
DNAse hypersensitivity at eeSNPs shows greater allele specificity in HCM. *X* axis: rank of DHS read counts, *Y* axis: absolute log-ratio of read counts mapping to the two alleles at a SNP. SNPs from different methods are selected similarly to Fig. 5. The analysis was performed on a subset of SNPs that were heterozygous in the sample. The median "white" lines represent LOESS (local regression) for each method. Confidence intervals for each median line is estimated using bootstrapping and are represented either by thin lines representing the LOESS of each bootstrap or by coloured shades representing confidence intervals in terms of standard deviation of bootstraps. Note the allele-specificity at SNPs detected by eQTeL and eqtnminer remains the same even if we control for number of SNPs per gene.

To ascertain the functional role of eeSNPs, we checked whether the change of a SNPs allele would affect their regulatory properties (such as protein binding, histone modifications and so on). For each cell line, we selected heterozygous SNPs by inspecting genotyped data or pooled reads from different histone modifications, DNAse-seq and CTCF. We first assessed allelic differences in footprint reads for human cardiac myocyte (HCM) (see Methods). As shown in Fig. 6, the eeSNPs that overlap a footprint show significantly greater (with odd-ratio of *M*=3.005 and *P* value <3.83 × 10^−17^) allele-specificity relative to SNPs identified by eqtnminer, consistent with eeSNP having a regulatory impact (allele-specifity comparison with LASSO is shown in Supplementary Fig. 10). For eeSNPs, we obtained 6.57-fold more reads mapping to the allele with more DNA-seq reads compared with the other allele (for eqtnminer, the average read difference was 1.8). We also found higher allele specificity for eeSNPs in other heart cell lines (Supplementary Fig. 11, HCF, SKMC) for DNASe-Seq reads. The trend of higher allelic specificity is also true in heart cell lines for histone modification H3K4me3, which is associated with active enhancers (Supplementary Fig. 12). Allele-specificity of eeSNPs suggests that they may underlie population variance in gene expression.

### eeSNPs are spatially proximal to their target gene

**Figure 7:**
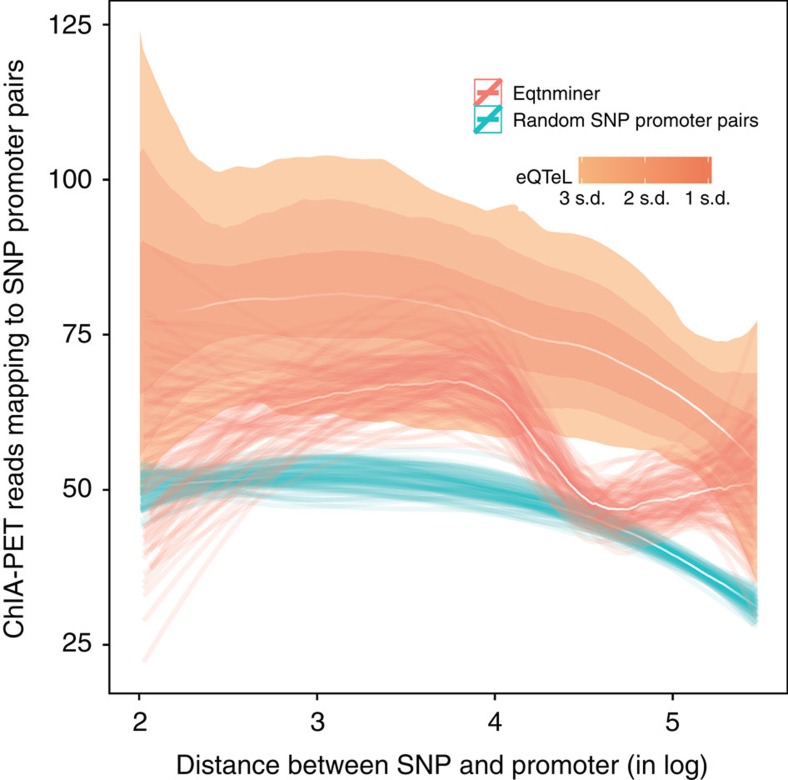
eeSNP-gene pairs are spatially proximal. X axis: the rank of eeSNP-gene distance (log 10), Y axis: ChIA-pet support. SNPs from eQTeL and eqtnminer are selected as in Fig. 8. The random SNP-gene pairs were selected so as to have the same distance distribution as for eeSNPs. SNP-gene pair closer to 100 bps were excluded. The median ‘white' lines represent LOESS (local regression) for each method. Confidence was estimated for each method just as in Fig. 6.

The spatial proximity of eeSNP with its target promoter is a pre-requisite for cis-regulation. Spatial proximity has been experimentally determined using chromatin interaction analysis with paired-end tags (ChIA-PET) assays^32^. Identified SNPs that were closer than 100 bps from their target promoters were excluded. We quantified spatial proximity of each eeSNP and its target by the number of pair-end reads supporting the proximity, whereby one of the reads overlaps with the target promoter and other read overlaps with the eeSNP. Analysis of pooled ChIA-PET data from various cell types suggests that, relative to controls, eeSNPs are significantly more proximal to their target genes (Fig. 7). This implies that eeSNPs are more likely to be cis-regulators of their target genes.

### eeSNPs disrupt motifs of cardiac transcription factors

**Figure 8:**
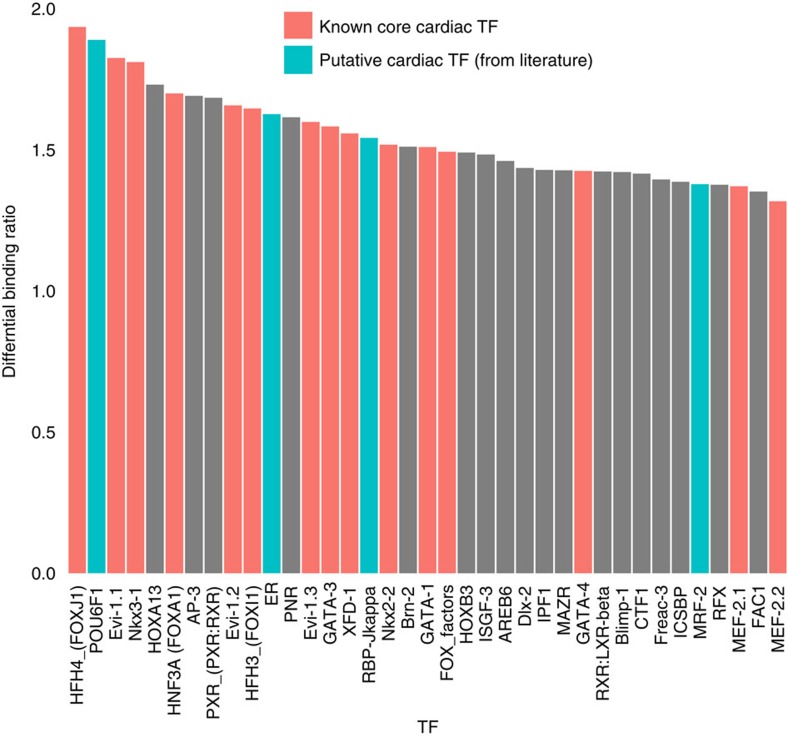
Regulatory motifs disrupted by eeSNP include several cardiac TFs. Only the motifs with average allele-specific binding score ratio>1.5 and Wilcoxon test *P* value<0.05 are shown, ordered by the ratio. Motifs corresponding to known cardiac TF families are shown in red and additional motifs with literature evidence of involvement in cardiac development or function are shown in blue.

A likely mechanism by which a regulatory SNP may affect gene expression is by disrupting binding of specific transcription factors^33^. For each of the 981 vertebrate TF motifs annotated in the TRANSFAC database^34^, we quantified (see Methods) the TF binding score difference between two alleles of eeSNP. We only considered the SNPs for which the score was significant for at least one of the alleles. As shown in Fig. 8, the core cardiac TF motifs (such as FOX, NKX, GATA) are among the TF binding motifs that are most likely to be disrupted by eeSNPs. This observation indicates that functional consequence of regulatory SNP might be heart specific. The disruption of STAT, MEF2, FOX, NKX and GATA transcription factor families are known to play important role in cardiovascular diseases^6^,^35^,^36^,^37^. This suggests that identified eeSNPs may have a specific transcriptional role in the heart.

### Proportion of eeSNPs that are causal

In the absence of extensive experimental data, it is difficult to estimate the proportion of eeSNPs that are causal. However, similar to a previous approach^11^, we used the proportion of eeSNPs that disrupt potential TF binding relative to the same for high-confidence putatively causal SNPs, as an independent estimate of proportion of eeSNPs likely to be causal (see Methods). Based on each TF motif, that was found to be preferentially disrupted by eeSNPs above, the proportion of eeSNPs estimated to be causal varied from 17 to 93%, with a mean estimate of 58% (Methods, Supplementary Fig. 12). Lastly, based on mammalian conservation data, we found that eeSNPs are more conserved than control SNPs (Supplementary Fig. 13).

## Discussion

Here we have introduced a novel Bayesian approach, eQTeL, that integrates genetic and epigenetic data in a statistically consistent manner to identify putatively causal genetic variants underlying the expression variance. We have shown that (i) eQTeL identifies combinations of SNPs (eeSNPs) that, compared with other methods, explain substantially greater portion of expression variability, (ii) eQTeL is especially effective in identifying SNPs with small effect sizes, (iii) 58% of the identified eeSNPs are likely to be causal, (iv) eeSNPs can predict sample specific expression much more accurately, (v) eeSNPs are much more likely to be bound by a regulatory factor in an allele-specific manner, (vi) eeSNPs preferentially disrupt core cardiac transcription factor binding and (vii) eeSNPs tend to be spatially proximal to their target genes. Taken together, our results strongly suggest that eQTeL captures a substantial proportion of putative causal regulatory genetic determinants underlying transcriptomic variance.

It is important to note limitations of eQTeL. First, eQTeL can only detect cis-eQTL and not trans-eQTL. Second, like other model-based association methods, eQTeL's computational speed is a bottleneck; however, using parallel cores and certain reasonable compromises in parameter estimation procedure, the computational burden can be substantially reduced. Third, eQTeL assumes normality of expression data, therefore the expression data needs to be pre-processed accordingly,which can be particularly problematic for certain kinds of high throughput data. Fourth, eQTeL can only detect SNPs with small effect size if they have high regulatory potential. Finally, eQTeL statistically infers the potentially causal SNPs and further experimental validations are required to establish causality.

eQTeL can effectively resolve LD and discriminate putative regulatory SNPs from myriad associated SNPs. This lays a foundation for future experimental studies to characterize genetic variants underlying disease risk. Finally, eQTeL can be extended by integrating additional layer of molecular data—easily achieved in Bayesian framework—to directly infer SNP that causes disease.

## Methods

### Modelling regulatory-interaction potential

There are R_1_ epigenetic features **E**_i_ that were used to predict if a SNP i lies in a regulatory region. In addition, we also have R_2_ interaction features **P**_ij_ that are predictive of the interaction between SNP i and gene *j*. We refer to a SNP that has high regulatory potential and high interaction potential as interacting-regulator, regardless of whether it associates with gene expression. Further, if the SNP is associated with gene expression, we refer to that SNP as *expression-regulator*. In our eQTeL approach, we model the regulatory-interaction potential *θ*_ij_ between SNP i and gene *j* as a combined function of epigenetic features **E**_i_ and interaction features **P**_ij_. Specifically, we use a Bayesian logistic regression model:


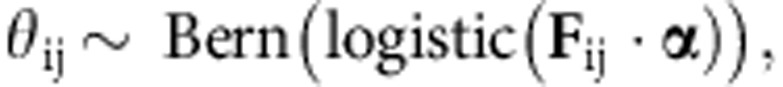


where **F**_ij_ is a concatenated set of features consisting of both **E**_i_ and **P**_ij_, and Bern is the Bernoulli distribution. The coefficients **α** are shared across all genes.

### Modelling gene expression

In our model, the expression of gene *j* depends not only on the allele status of candidate SNPs, but also on the estimated regulatory-interaction potential of the SNP *i* and gene *j* pair. Specifically, given gene expression in n samples **Y**_j_=(Y_j1_,…,*Y*_jn_), we model the vector of expression **Y**_j_ for gene *j* as a linear function of the allele status for all candidate SNPs, **X**={X_1_,⋯, X_p_} where X_i_ is allele status of SNP i over the *n* samples:


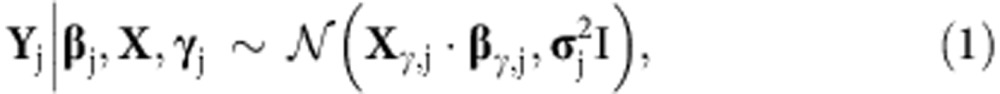


where the effect β_ij_ of SNP i on the expression of gene *j* is non-zero only when indicator variable *γ*_ij_=1. In other words, *γ*_ij_=1 signifies whether SNP i is associated with the expression of gene *j*. **X**_**γ**,j_ (and **β**_**γ**,j_) refers to a subset of SNPs for which *γ*_ij_=1.

If a SNP lies within a genomic region that is deemed to be (i) a regulator, and (ii) interacting with the target gene, then the SNP is likely to affect the gene's expression. Thus, the regulatory-interaction potential for each pair of SNP i and gene *j* enters our gene expression model through the prior distribution on the indicator variables γ_ij_,


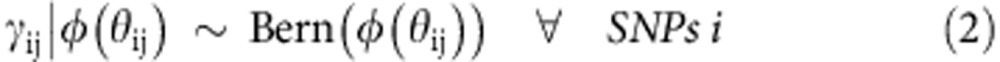


where the function *ϕ*(*θ*) is defined so that 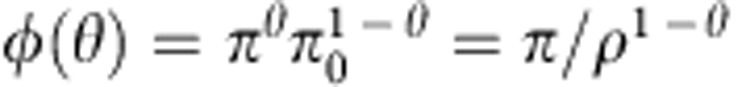 with *π* being our prior probability for each SNP to be expression-regulator and let *π*_0_=*π*/*ρ* be the prior probability when the SNP does not reside in such a region, where *ρ* is an amplification factor. An uniform prior for *π*∈(*m/e*, *M/e*) is defined, where *m* and *M* are respectively the minimum and the maximum number of expected expression-regulators. However, no substantial difference in results was observed when we just fixed 
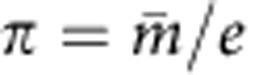
 where m is expected number of expression regulators. A value of *ρ*=100 was used because performance of model was insensitive to choice of *ρ*∈(100, 1,000).

Because of severe multiple testing corrections, association studies miss many potential causal regulators that have relatively small effect on expression. In our eQTeL model, overall sparsity is controlled by two factors: (a) the fraction of SNPs which are interacting-regulators, that is, *E*(*θ*) and (b) the fraction of interacting-regulators which are expression-regulators, that is, *π*. This is because the overall sparsity is a product of the two factors, that is, log*E*(*ϕ*(*θ*))≈*E*(*θ*)log*π* assuming *ρ*>>>1. Thus, the effective sparsity constraints are less conservative on SNPs that lie within an interacting-regulator in our eQTeL model, which allows us to capture potential causal expression-regulator SNPs with small (but non-zero) effects on expression variance (Fig. 4 and Supplementary Fig. 6; refer to Supplementary Note 5).

We also employ a standard prior distribution, Zeller's g-prior^20^, for our linear model parameters,


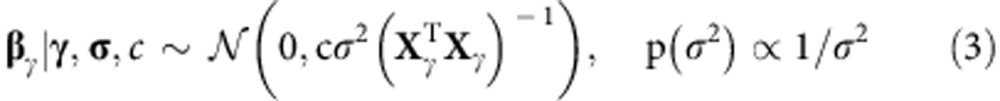


and we also define the following prior distributions for the rest of the parameters as


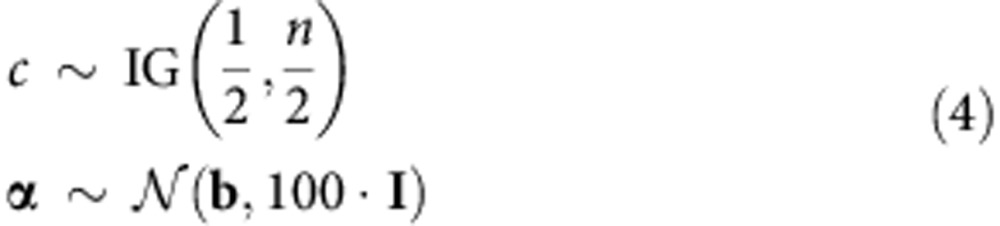


The first element of **α**, *α*_0_ is the bias term, and **b** is the prior for **α**, and is set to 0, except for b_0_ (the prior for *α*_0_), which can be used to control the sparsity on the number of interacting-regulators. We expect 1% of all SNPs to be regulators. To achieve this level of sparsity in number of regulators, *b*_0_ was set to log(*e*/(*p*−*e*)), where e is expected number of interacting-regulators, and was set to *p*/100. That is, *b*_0_=log(1/99).

Refer to Supplementary Note 1 for the eQTeL's inference algorithm, initialization and convergence criteria.

### Cardiac expression data (MAGNet)

Samples of cardiac tissue (*n*=313) were acquired from patients from the Myocardial Applied Genomics Network (MAGNet; www.med.upenn.edu/magnet). Left ventricular free-wall tissue was harvested at the time of cardiac surgery from subjects with heart failure undergoing transplantation and from unused donor hearts. Genomic DNA was extracted using the Gentra Puregene Tissue Kit (Qiagen, CA) according to manufacturer's instructions. Total RNA was extracted using the miRNeasy Kit (Qiagen) including DNAse treatment. RNA concentration and quality was determined using the NanoVue Plus spectrophotometer (GE Healthcare) and the Agilent 2,100 RNA Nano Chip (Agilent). To assess gene expression, RNA was hybridized with Affymetrix Genechip ST1.1 arrays using manufacturer instructions. CEL files were normalized with the robust multi-array analysis using the oligo package in Bioconductor^38^. To remove potential batch effects, expression values were further adjusted using ComBat, an empirical Bayes method that estimates parameters for location and scale adjustment of each batch for each gene independently^39^. Probe sets were removed if they displayed robust multi-array analysis expression values <4.8 on all arrays. This filtering yielded sets of genes present well above background levels in the human heart. Probeset showing no annotated cross hybridization potential were kept, leaving 15,395 probes for final analysis.

### Selection of genes

The genes were selected such that they had at least one significantly associated SNPs based on univariate-eQTL (Matrix eQTL). 1,880 genes were thus selected using FDR threshold of 1E-6 using Matrix-eQTL (Lappalanien *et al*.). We have no reason to believe that this selection is favourable to eQTeL.

### Pre-procession of gene-expression

It has been found that removing technical biases and confounding factors can greatly improve the association studies. Normalization of gene-expression data to remove confounding factors have been studied extensively (40,41). In association studies the comparison is across individual and not across genes, and therefore main aim of the normalization is to make the gene-expression distribution across samples comparable. Similar to Lappalainen *et al*., we use PEER^40^ to remove the confounding factors from expression data as pre-processing. Given expression data for multiple individuals, PEER identifies hidden factors that explain a large proportion of global expression variability. Factors represent covariates that affect multiple gene and are therefore most likely to be confounding factors or technical biases. The factors are then regressed out from the expression and residual are used for performing association studies. In certain cases, such in trans-eQTL, a genetic-factor can affect multiple SNPs and PEER might remove biologically relevant signal. However, since the aim of the paper is to identify cis-eQTL, that is, local effects, we can safely use PEER.

To determine number of factors (K) to be removed using PEER, we used approach similar to Lappalaninen *et al*. We ran PEER for 16,271 Affymetrix gene probes from MagNet using parameter K=0, 3, 5, 10, 15 and 20; then we compared number of genes (eGenes) that have at least one SNPs significantly associated with expression (*P* value<1 × 10^−6^). We chose *K*=10 because number of eGenes plateaued at *K*=10. Factors from PEER were regressed out from the expression and residual expression was used for further analyses.

Linear regression assumes normality of the expression data. We converted the residual data from PEER to standard normal distribution before performing the association analysis.

### Genotypes and imputation for cardiac samples

DNA samples were genotyped using Affymetrix Genome Wide SNP Array 6.0 and analysed per manufactures instructions. We applied quality control (QC) filters to exclude unreliable samples, samples with cryptic relatedness and samples that were not genetically inferred Caucasian. After QC filtering, 313 individuals remained. All analyses were conducted using software package PLINK^14^. For the analysis reported here, we eliminated SNPs with genotype call rate <95%, with minor allele frequency (MAF) < 15%, or if there was significant departure from Hardy–Weinberg equilibrium (*P*<10^−6^). A total of 360,046 SNPs passed QC and were available for analysis. To improve cross study comparisons, genotype imputation was performed using the Minimac (v 2012.11.16) (ref. 27) program. Imputation results were filtered at an imputation quality threshold of 0.5 and a MAF threshold of 0.15.

PLINK^14^ was used to infer LD block for the genotypes. Default setting of SNPs within 200 Kb was used to estimate it.

### Epigenetic data and interaction features

Epigenetic data were obtained from ENCODE, Roadmap epigenome project and GEO database for following heart tissues: AoAF, HCM, HCF, fetal-hearts, adult-hearts, left ventricle, right ventricle, arota, and right atrium. Because DNAse I footprints were used to validate eeSNPs, they were excluded from the feature importance (**α**) estimation of eQTeL. Supplementary Fig. 2 lists the epigenetic and interaction features, that were critical for identification of interacting-regulators. We assessed the importance of epigenetic factors directly overlapping each SNP within 50 bps flanking region (suffix .50 in Supplementary Fig. 2). We also assessed the importance of epigenetic factors in broader context of each SNP within 500 bps flanking region (suffix .500 in Supplementary Fig. 2). Interaction features between a gene-promoter and a region containing SNP were calculated using RNASeq and DHS data from 15 cell types (A549, Bj, H1hesc, Hepg2, Hsmm, K562, Nhek, Ag04450, Gm12878, Helas3, Hmec, Huvec, Mcf7, Nhlf and Sknshra). These features include: (a)correlation and absolute correlation between DHS of the region and DHS of the promoter (b) correlation and absolute correlation between DHS of the region and RNASeq FPKM of the gene.

Both epigenetic and interaction features were normalized to mean of 0 and standard deviation of 1. This implies that distribution of each of these features for a set of random SNPs were expected to have 0 mean and 1 s.d. Therefore, *y* axis in Supplementary Fig. 2 shows absolute enrichment over random-SNPs with units in s.d.

### Estimating fraction of putatively causal eeSNP

Using an approach similar to Lappalanien *et al*.^11^, we estimated proportion of eeSNP that are putatively causal. Clearly, an independent estimation of proportion of causal SNPs cannot rely on features used to identify eeSNPs, or any other potentially correlated feature, such as footprints. Thus, for an independent estimate of the proportion of causal SNPs, we used potential TF binding disruption by a SNP allele. Following Lappalanien *et al*., using Matrixeqtl^25^, we first identified causal SNPs as follows. For each gene we identified best and second best associated SNPs, and the best SNP was deemed causal if (i) the best SNP association was significant (FDR<10^−6^) and (ii) the difference in association score (−log10 *P* value) between the best and the second best SNPs was greater than a threshold (conservatively, 2.5, a la Lappalanien *et al*.).

For each TF motif, we obtained the disruption at each SNP (decrease in motif match scores due minor allele relative to major allele) thus obtaining two distributions, one for causal SNPs and another for the presumed non-functional background. Using distribution of motif disruption score for causal SNP, we identified TF motifs that are preferentially disrupted by causal SNPs. For each of such motif y, we calculated an enrichment score *c*_causal,y_ which is the ratio of means of TF motif disruption score between the causal and a set of presumed non-causal SNPs. For motif *y*, we similarly calculated the enrichment score for eeSNPs *c*_eeSNP,y_. Following Lappalainen *et al*., we then estimated the fraction of eeSNPs likely to be causal as 
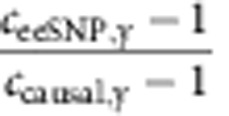
. Supplementary Fig. 14 shows these proportion of eeSNP that is likely to be causal for all selected motifs, suggesting that overall 58% of eeSNPs are putatively causal.

Functional explained variance and expression predictability was defined as explained variance by subset of expression-regulators that mapped to a DNAse I footprint.

### Simulation study

Simulation was done on 200 genes. We used 174,800 SNPs (874 SNPs per each gene) for 313 samples from MAGNet genotype data. 1% of total SNPs were declared as enhancers. We estimated, number of causal regulatory SNPs and distribution of explained expression variance by genotype by running eQTeL in MAGNet data. Using estimated number of causal regulators from MAGNet, expression-regulators were selected among enhancer per gene. Effect-size of each expression regulator was generated from 
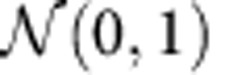
, that is finally being used to generate expression for each gene using a linear model. Finally a random noise was added such that explained variance by expression-regulators will be same as estimated from MAGNet data. For each regulator SNP, seven epigenetic features (DNAse, H3K4me1, H3K4me3, P300, H3K27me3, H3K36me3 and H3K9me3) for heart were generated from distribution derived from validated heart enhancers^6^. For all other SNPs epigenetic features were generated from random SNP background.

### Motif binding score differential

For each of the 981 vertebrate TF motif from TRANSFAC database^42^, we scanned the 50 bps flanking eeSNPs (and for 10,000 control SNPs randomly sampled from 300,000 SNPs) for the presence of motif using pwmscan tool^43^, separately for the major and the minor allele. Only the cases where at one of the two alleles had a motif hits (*P* value<0.0002) were further considered. For each such case, the difference in the binding score for the two alleles was computed, as the difference in log(*P* value). For each motif, the binding differential score for eeSNPs and the control SNPs were compared using Wilcoxon test and the motifs which had at least 1.5-fold greater differential among eSNPs and a *P* value<0.05 were identified.

### DNAse footprint enrichment

From^31^ we obtained a list of genomic locations, for 41 different cell-types, where significant evidence of *in vitro* protein binding event were detected using DNAse-footprint. For each tissue, we calculated fraction of number of SNP that have a footprint in the 50 bps flanking it.

### Allelic imbalance and ChIA-PET analysis

DNAse hypersensitivity (DHS-seq) reads for heart cells (HCM sample) were obtained and mapped to eeSNPs (and control SNPs). Heterozygousity at each SNP locus was ascertained by the presence of multiple alleles among the reads mapping to the SNP location. For each such locus, the allelic imbalance was calculated as the difference in the number of reads mapped to each allele. The allelic imbalance was plotted against the overall signal intensity rank.

ChIA-pet assay identified spatially proximal genomic regions where at least one of the region is bound by PolII. Because ChiA-pet data is unavailable for heart-related cell types, we pooled multiple ChiA-pet data from *K562, Hela, Nb4 and MCF7*. For each 50 bps flanking an eeSNP (or control SNP) and the target promoter pair, number of ChIA-pet reads supporting the spatial proximity of the two loci were recorded. The ChiA-pet support for each SNP-gene pair was then compared for different methods after controlling for the genomic distance between the SNP and its target gene.

In Figs 6 and 7, median ‘white' lines represent LOESS (local regression) for each method. Confidence interval for each median line is estimated using bootstrapping and they are shown in the s using either of following two ways: by thin lines representing LOESS of each bootstrap, or by coloured regions representing confidence intervals in terms of standard deviation of bootstraps.

### Software availability

The implementation of eQTeL with its source code is freely available at (www.cbcb.umd.edu/software/goal) as a R-package under MIT license.

For details of other eQTL methods (Supplementary Note 3); expression explained variance and predictability (Supplementary Note 6); and scalability of eQTeL (Supplementary Note 7) refer to Supplementary Notes.

## Additional information

**How to cite this article:** Das, A. *et al*. Bayesian integration of genetics and epigenetics detects causal regulatory SNPs underlying expression variability. *Nat. Commun.* 6:8555 doi: 10.1038/ncomms9555 (2015).

## Supplementary Material

Supplementary Information Supplementary Figures 1-15, Supplementary Notes 1-7 and Supplementary References

Supplementary Data 1 Regulatory SNP in MAGNet detected by eQTeL

Supplementary Data 2 Replicated eQTL between MAGNet and GTEx
