# Supplementary material for: Bayesian integration of genetics and epigenetics detects causal regulatory SNPs underlying expression variability
Source: Nat Commun. 2015 Oct 12;6:8555. doi: 10.1038/ncomms9555 (PMC4633824; doi:10.1038/ncomms9555)
Supplement: Supplementary Information — Supplementary Figures 1-15, Supplementary Notes 1-7 and Supplementary References [file ncomms9555-s1.pdf]

Supplementary Figures

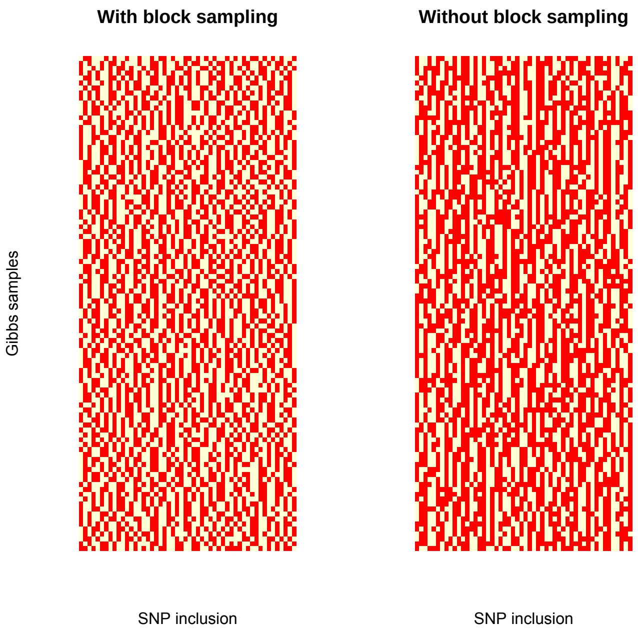

Fig. 1: Mixing rate of eQTeL with and without block sampler. (Note: regulatory and interaction priors were removed for this exposition). The block sampler leverages information of Linkage disequilibrium (LD) blocks to choose sparse set of SNPs within each LD block. In the current example, there are two (identical) SNPs within each LD block. The sampler without block sampler are more often stuck at previously selected SNPs in consecutive MCMC iterations compared to the block sampler. This problem will exponentially increase with growing number of SNPs in LD block. On the other hand, block sampler chooses subset of SNPs with a LD from their full posterior distribution in each iteration independently using a MH sampler. Relatively higher number of combinations of SNPs will be explored by block sampler. The block sampler chooses comparatively better subset of SNPs since it explores relatively larger fraction of the model space.

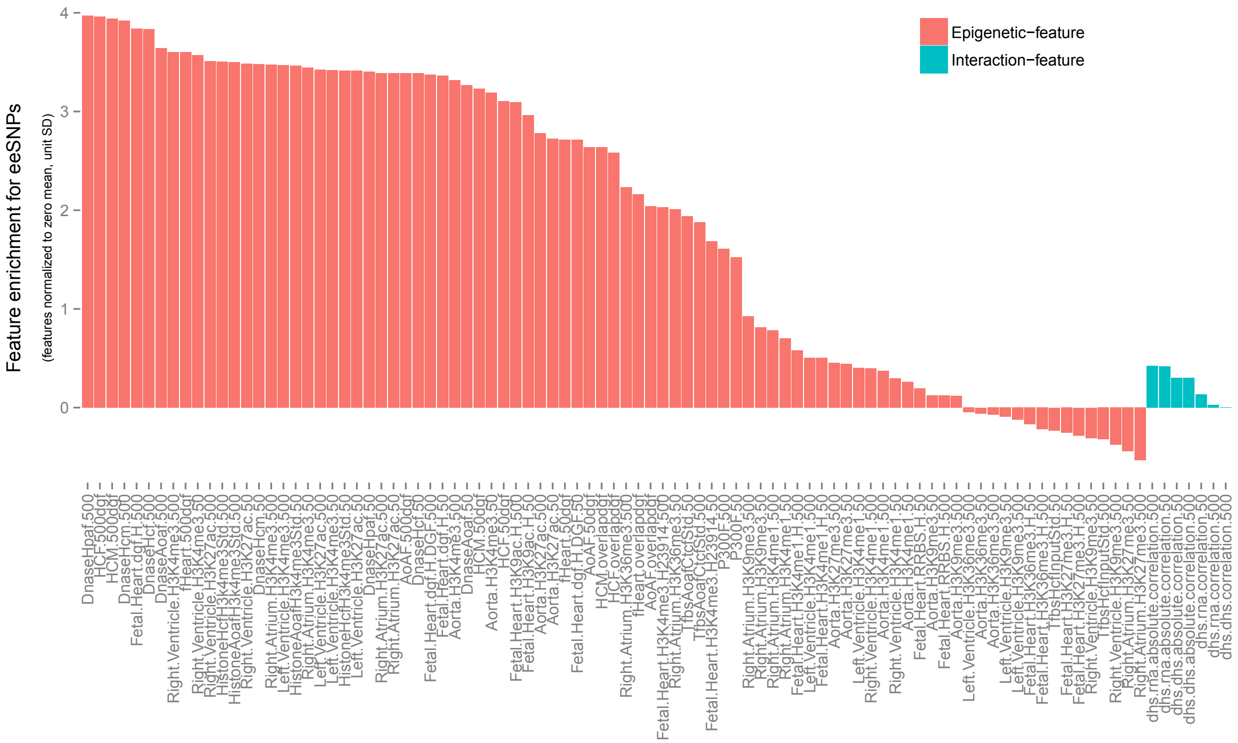

Fig. 2: Feature-analysis: Significant features ($p - value < 10^{-6}$) are sorted by their enrichment in eeSNPs relative to random SNPs (Note: features are not independent). Lists the epigenetic and

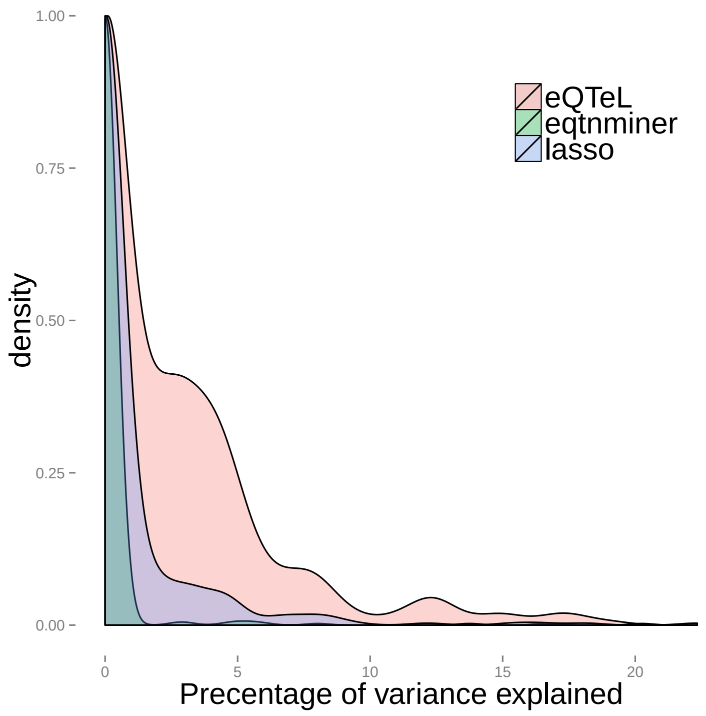

Fig. 3: Validation of eeSNPs in GTEx: Comparative performance of SNPs detected by eQTeL, LASSO and eqtnminer in terms of explained variance. Number of SNPs were controlled for each method (as in Fig 2). SNPs from eQTeL were selected using posterior probability > 0.5. The figure shows (5 fold) cross-validated explained variance and correlation between predicted expression using alleles of identified SNPs for each methods.

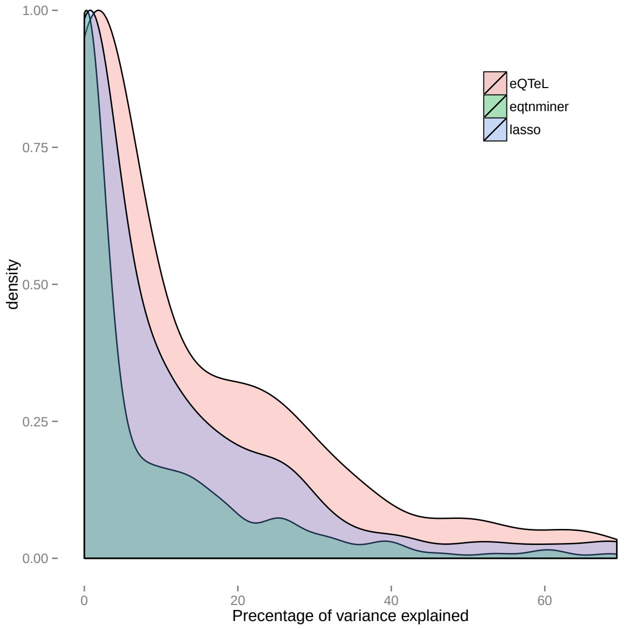

Fig. 4: Comparative performance of eQTeL in terms of explained variance in simulated data: Number of SNPs were controlled for each method (as in Fig 2). SNPs from eQTeL were selected using posterior probability > 0.5. SNPs from eQTeL were identified with posterior probability > 0.5. The figure shows (10 fold) cross-validated explained variance for each method.

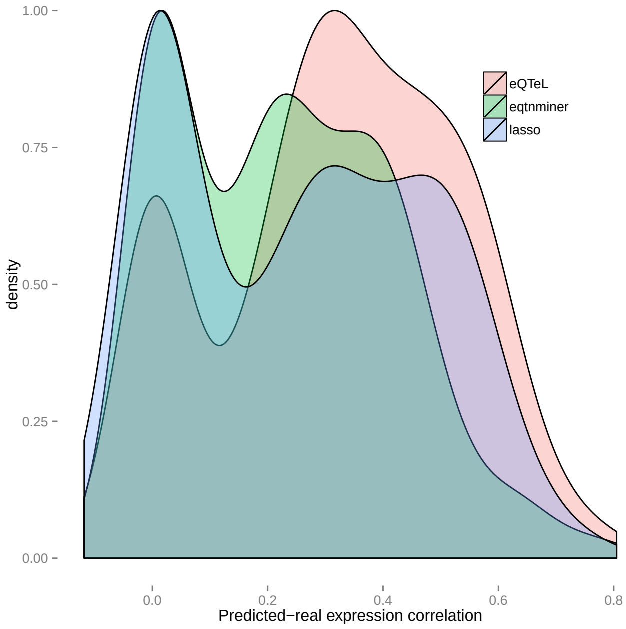

Fig. 5: Comparative performance of eQTeL in terms of expression predictability in simulated data: Number of SNPs were controlled for each method (as in Fig 2). SNPs from eQTeL were selected using posterior probability > 0.5. The figure shows (10 fold) cross-validated correlation between predicted expression using alleles of identified SNPs for each method.

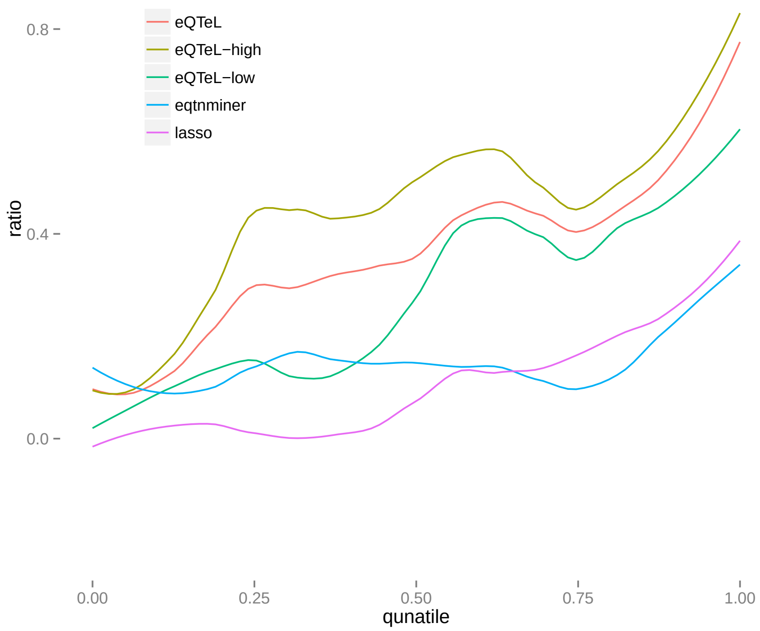

Fig. 6: Comparison of recall-rate of different methods (controlled for overall effective sparsity). eQTeL-high is eeSNPs with high regulatory potential (above 75 quantile). eQTeL-low is eeSNPs with low regulatory potential in lower 25% quantile.

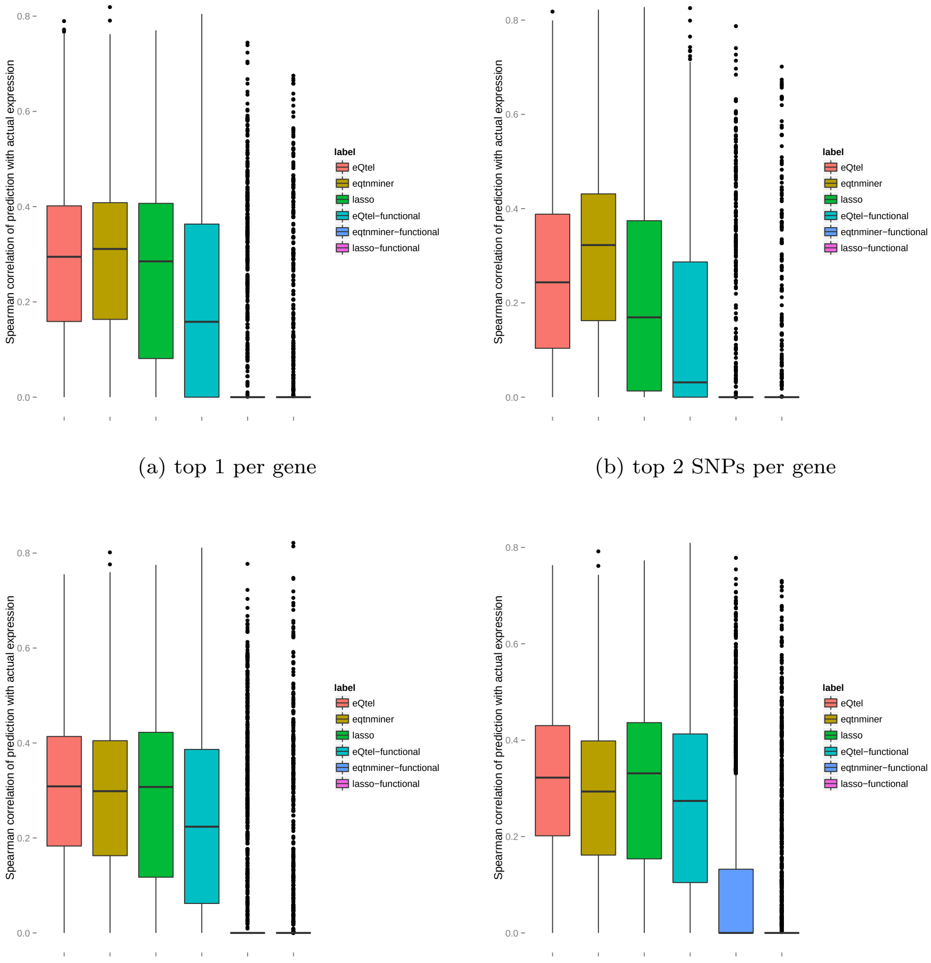

(a) top 1 per gene

(b) top 2 SNPs per gene

//

(c) top 3 SNPs per gene

(d) top 5 SNPs per gene

Fig. 7: Comparative performance of eQTeL as number of SNP per genes are increased in imputed data.

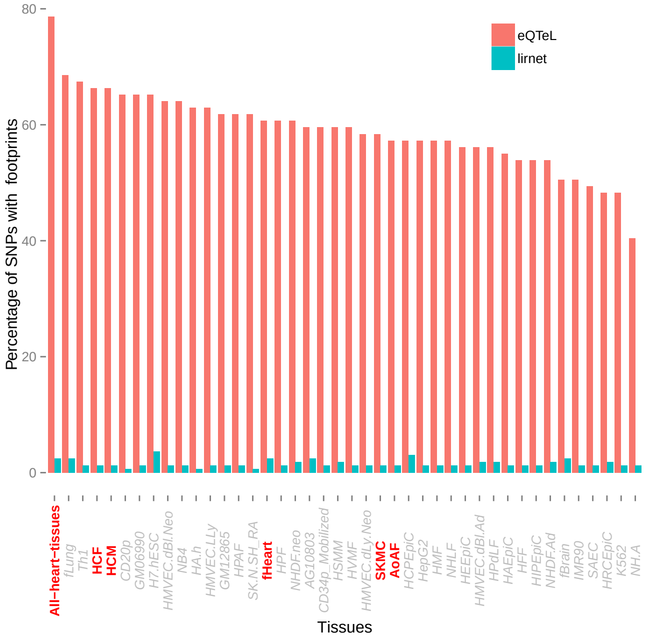

Fig. 8: Lirnet enrichment of DGF footprint: This analysis is based on 162 SNPs identified by eQTeL and Lirnet. We analyzed footprint in 42 cell lines from Neph et. al. overlapping the SNP within 25 bps the SNP loci by using bedtools for each of the method. The heart-related-tissues are highlighted in red in the figure. The left-most bar represents pooled data from all heart-related cell types.

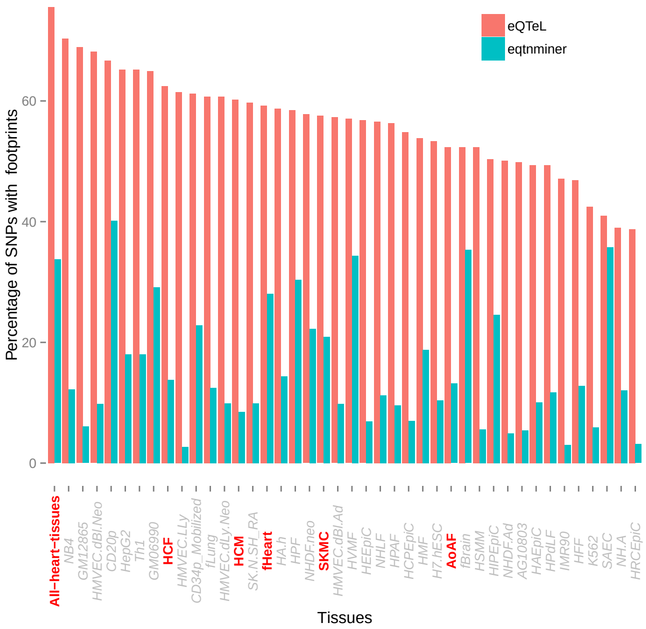

Fig. 9: Eqtnminer subset selection. The eqtnminer with 8 dimensional features (from 95 dimensional features), selected based on feature importance estimated by eQTeL. Non-redundant features were chosen. The performance of eqtnminer improves substantially compared to 95 dimensional eqtnminer.

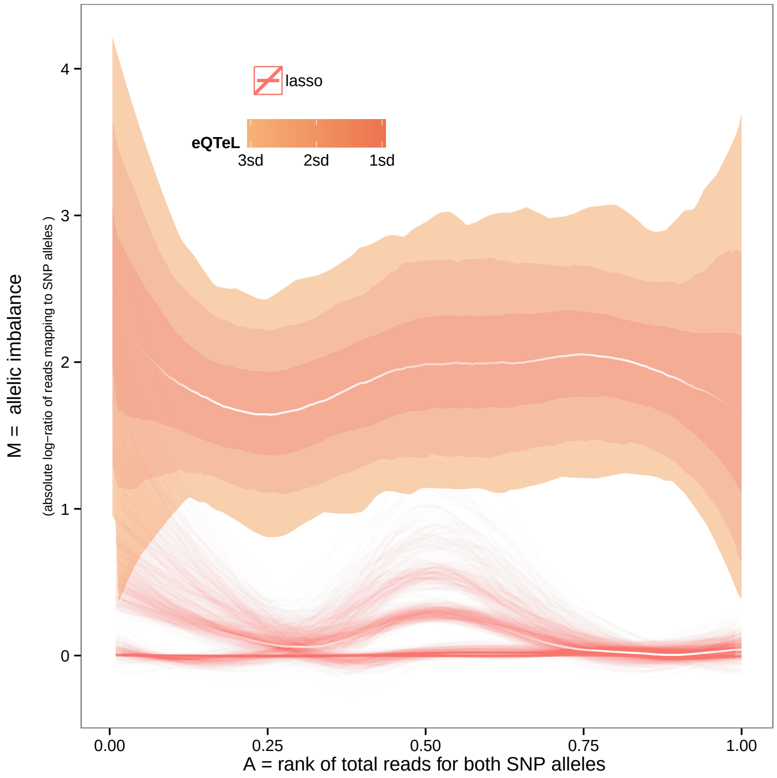

Fig. 10: DNAse hypersensitivity at eeSNPs shows greater allele specificity in HCM. X axis: rank of DHS read counts, Y axis: absolute log-ratio of read counts mapping to the two alleles at a SNP. SNPs from different methods are selected similar to Fig 5. The median white lines represent LOESS (local regression) for each method. Confidence interval for each median line is estimated using bootstrapping and are represented either by thin lines representing LOESS of each bootstrap, or by colored shades representing confidence intervals in terms of standard deviation of bootstraps. Note the allele-specificity at SNPs detected by eQTeL and eqtnminer remains same even if we control for number of SNPs per gene.

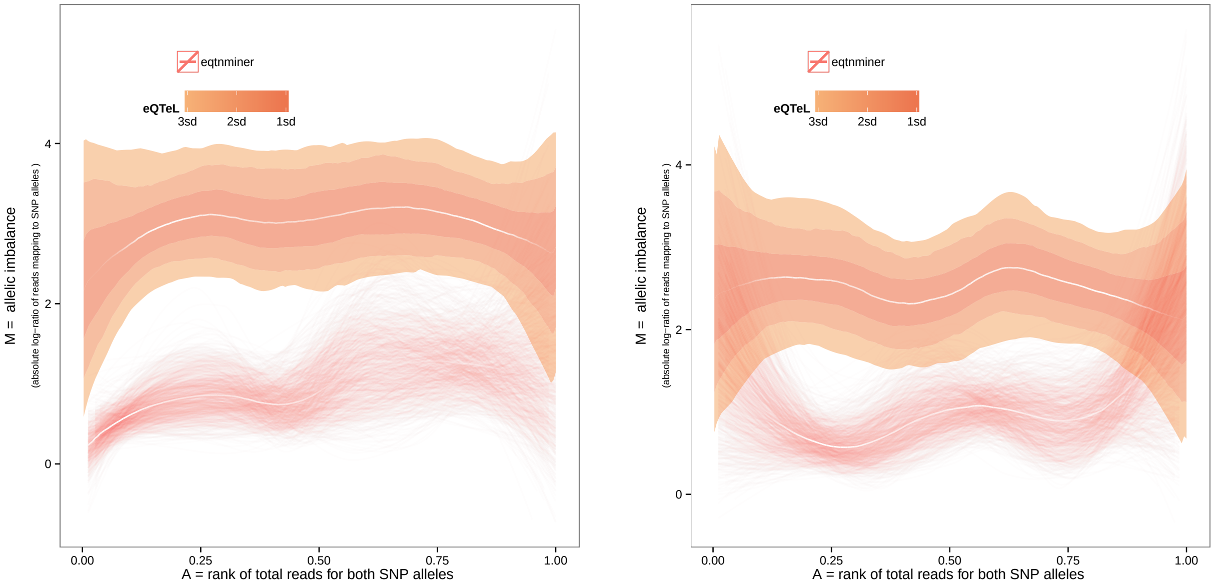

(a) SKMC

(b) HCF

Fig. 11: Relative allele specificity (in terms of DHS reads) by SNPs identified by different methods: X axis: rank of DHS read counts, Y axis: absolute log-ratio of read counts mapping to the two alleles at a SNP. SNPs from different methods are selected similar to fig 5. The median white lines represent LOESS (local regression) for each method. Confidence interval for each median line is estimated using bootstrapping and they are shown in the figures using either of following two ways: by thin lines representing LOESS of each bootstrap, or by colored shades representing confidence intervals in terms of standard deviation of bootstraps. Note the allele-specificity at SNPs detected by eQTeL and eqtnminer remains same even if we control for number of SNPs per gene.

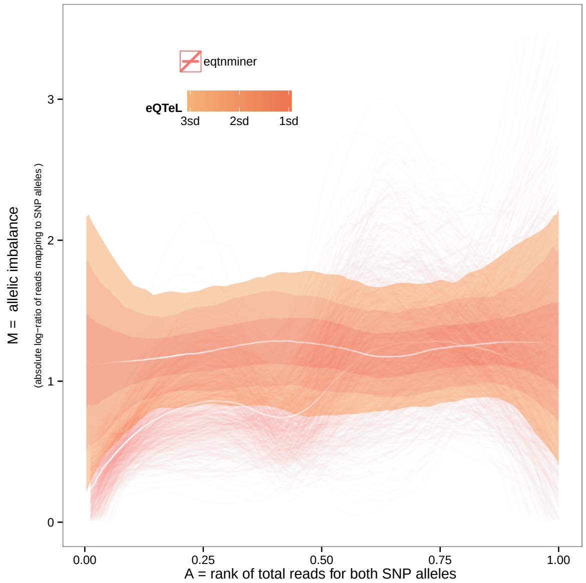

Fig. 12: Relative allele specificity by SNPs (in terms of H3K4me3) identified by different methods: X axis: rank of DHS read counts, Y axis: absolute log-ratio of read counts mapping to the two alleles at a SNP. SNPs from different methods are selected similar to fig 5. The median white lines represent LOESS (local regression) for each method. Confidence interval for each median line is estimated using bootstrapping and they are shown in the figures using either of following two ways: by thin lines representing LOESS of each bootstrap, or by colored shades representing confidence intervals in terms of standard deviation of bootstraps. Note the allele-specificity at SNPs detected by eQTeL and eqtnminer remains same even if we control for number of SNPs per gene.

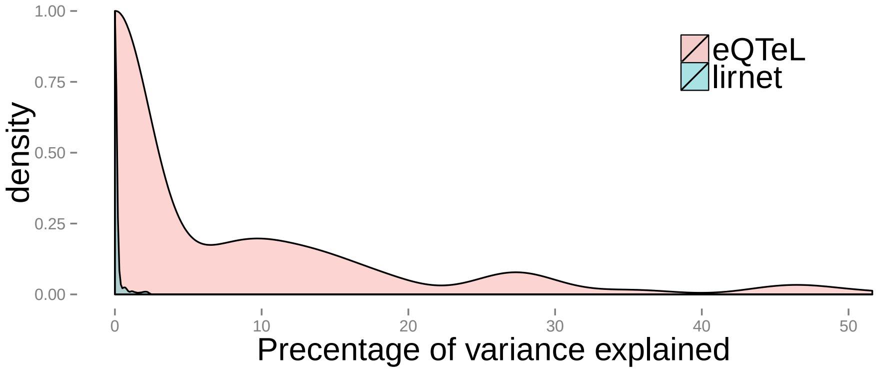

(a) Explained variance

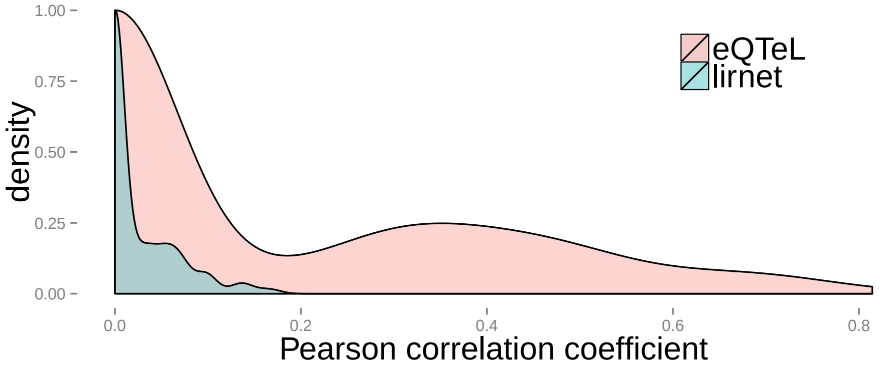

(b) Expression predictiblity

Fig. 13: Comparative performance of Lirnet: Comparative performance of Lirnet in terms of explained variance and expression predictability for 200 genes. Number of SNPs were controlled for each method (as in Fig 2). SNPs from eQTeL were selected using posterior probability > 0.5. The figure shows (10 fold) cross-validated correlation between predicted expression using alleles of identified SNPs for each method.

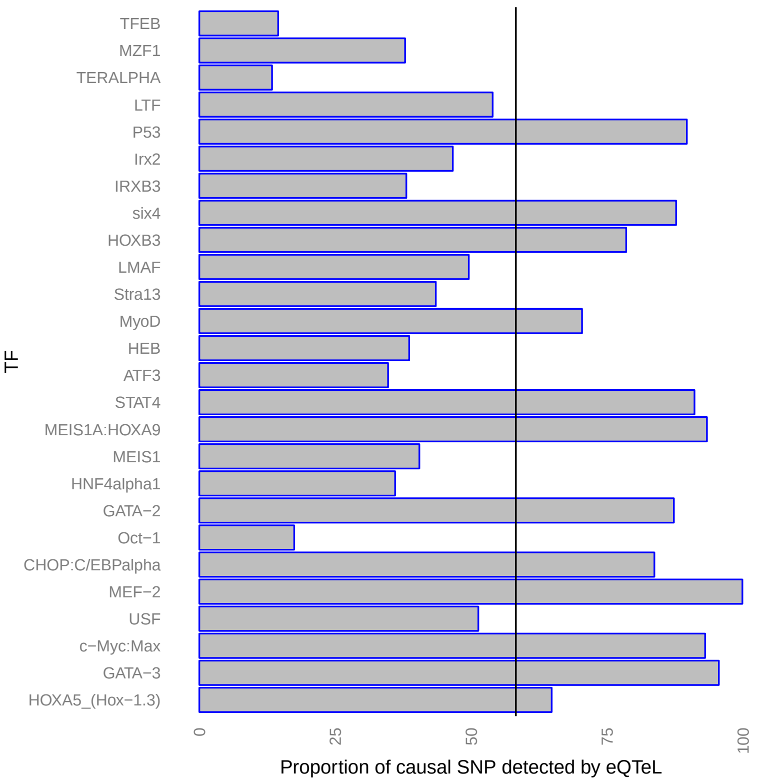

Fig. 14: Proportion of causal SNPs detected by eQTeL: Highly putatively *causal* were identified SNPs using difference in association between best-associated SNP and second-associated SNP for each gene. Y axis shows mammalian TF motifs that are preferetially disrupted by *causal* SNPs. For each of these motifs, proportion of causal SNPs among eeSNPs was estimated using ratio of relative enrichment (over background) of motif disruption score (differential binding score between major allele and minor allele of SNP) between eeSNPs and *causal SNPs*.

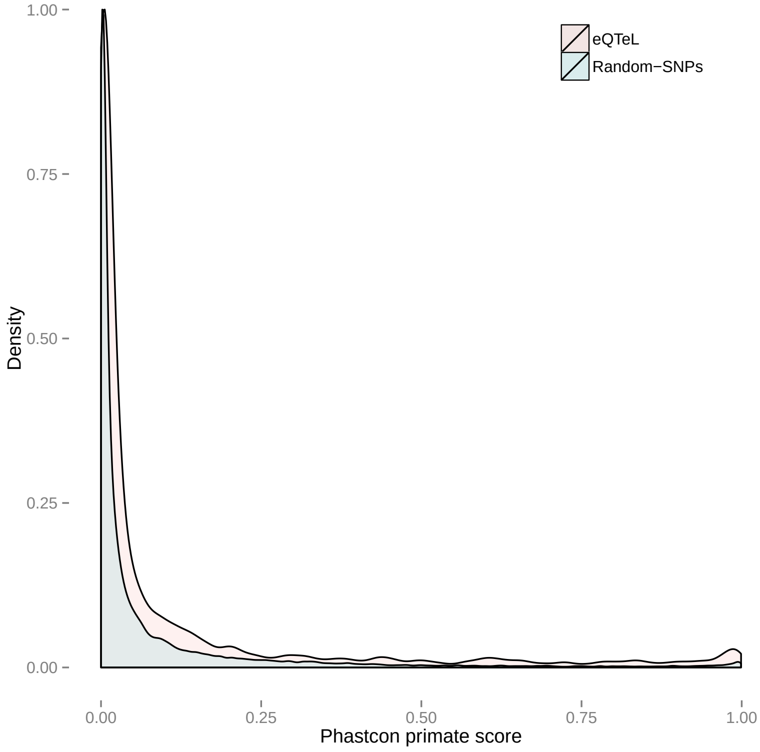

Fig. 15: Conseravation of eeSNPs. Distribution of mammalian PhasCons scores for eeSNPs and the control SNPs. The ratio of the two means is 1.49 and Wilcoxon test p-value $< 5 * 10^{-5}$.

Supplementary Notes

Supplementary Note 1: Inference

We used a combination of Gibbs and Metropolis-Hasting sampling [3] to jointly estimate the full posterior distribution of our model parameters.

1.1 Sampling γ parameters accounting for Linkage Disequilibrium

We estimated linkage disequilibrium block using PLINK [4], by using default setting of SNPs within 200kb. The effects of SNPs in Linkage Disequilibrium are dependent on each other because the SNP alleles are highly correlated. Gibbs or Metropolis-Hastings samplers that ignore the LD structure of SNPs can get stuck in local minima while failing to explore high probability combinations of $\boldsymbol{\gamma}$ (Fig. S15). To overcome these poor mixing properties, we devise a block MCMC sampler that explicitly uses LD-block information to sample from the posterior probability of a LD-block i.e.

$$p(\boldsymbol{\gamma}_{LD}|.) = \frac{P(\mathbf{Y}|\boldsymbol{\gamma}_{LD}, \boldsymbol{\gamma}_{-LD}) \prod_i p(\gamma_i|\theta_i)}{\sum_{\gamma'_{LD}} P(\mathbf{Y}|\boldsymbol{\gamma}'_{LD}, \boldsymbol{\gamma}_{-LD}) \prod_i p(\gamma'_i|\theta_i)}$$

where, $\boldsymbol{\gamma}_{LD}$ and $\boldsymbol{\gamma}_{-LD}$ are γ of set of SNPs respectively within and outside the LD-block. The resulting sampler mixed much faster (Fig. S15) by exploring high probability models in a hierarchical fashion: we use a Gibbs sampler to sample highly-probable combinations of LD blocks and within these sampled LD block, and then a Metropolis-Hasting sampler is used to sample a sparse combination of SNPs that explain expression variance.

1.2 Sampling α and θ parameters

We follow the latent variable Gibbs sampling strategy of [5] to sample the logistic regression parameters $\boldsymbol{\alpha}$. Specifically, we can sample latent variables from a Pólya-gamma distribution,

$$w_i|\boldsymbol{\alpha} \sim \mathcal{PG}(1, E_i\boldsymbol{\alpha}) \tag{1}$$

and then sample $\boldsymbol{\alpha}$ from a normal distribution,

$$\boldsymbol{\alpha} \sim N(m_w, V_w)$$

where, $V_w = (\mathbf{F}^T\Omega\mathbf{F} + B^{-1})^{-1}$, $m_w = V_w(\mathbf{F}^T\kappa(\boldsymbol{\theta}) + \mathbf{B}^{-1}\mathbf{b})$ with $\kappa(\boldsymbol{\theta}) = (\boldsymbol{\theta} - .5)$ and Ω being a diagonal matrix of the w_i's. Then, for each SNP i and gene j, the regulatory-interaction potential θ_{ij} is sampled from its posterior distribution as

$$P(\theta_{ij} = 1) = \frac{\phi(\gamma_{ij})\text{logistic}(E_i\boldsymbol{\alpha})}{\phi(\gamma_{ij})\text{logistic}(E_i\boldsymbol{\alpha}) + (1 - \phi(\gamma_{ij}))(1 - \text{logistic}(E_i\boldsymbol{\alpha}))} \tag{2}$$

where $\phi(\gamma) = \pi^{\gamma}\pi_0^{1-\gamma}$. If θ were estimated based only on whether the corresponding SNP was an expression-regulator (i.e based on value of γ), then the resulting estimation of regulatory-interaction potential would be equivalent to supervised learning. On the other hand, if θ were sampled on posterior that depended only on current estimate of $\boldsymbol{\alpha}$ and not on γ, the resulting estimation be equivalent to clustering. eQTeL, however, uses both in its posterior sample and therefore induces a semi-supervised clustering of genomic regions into interacting regulator and neutral regions. This approach to model θ induces a semi-supervised clustering of genomic-region into interacting-regulators and noninteracting-regulators, since each MCMC iteration produces a sample of θ_{ij} for each SNP that depends on its γ_{ij} in addition to its current estimate of regulatory and interaction potentials.

1.3 Inference of β, σ^2 and c

For simplifying the notations, in the section we only consider subset of SNPs which were selected by the model so that $\mathbf{X}$ represents $\mathbf{X}_\gamma$ (this is $n \times q$ matrix, where n is number of samples and q is total number of SNP selected in the model). The generative model for β, σ^2 and c are:

$$\begin{aligned}
Y|\boldsymbol{\beta}, X, \gamma &\sim N(X^T\boldsymbol{\beta}, \sigma^2 I) \\
\boldsymbol{\beta}|c, \sigma &\sim N(0, c\sigma^2(X^TX)^{-1}) \\
\sigma^2 &\sim IG(\nu/2, \nu\lambda/2) \\
c &\sim IG(\frac{1}{2}, \frac{n}{2})
\end{aligned} \quad (3)$$

For Zellner's g-prior ν is usually assumed to be zero. β, σ^2 and c are sampled from the full posterior distribution as:

$$\begin{aligned}
P(\beta, \sigma^2, c|Y, X) \propto\ & c^{3/2} exp(-n/2c) \\
& \sigma^{-2(\nu/2+1)}\sigma^{-n}(c\sigma^2)^{-q/2} \\
& exp\{-\frac{1}{2\sigma^2}\nu\lambda \\
& -\frac{1}{2\sigma^2}(Y - X\hat{\beta})^T(Y - X\hat{\beta}) \\
& -\frac{1}{2\sigma^2}(\beta - \hat{\beta})^T X^T X(\beta - \hat{\beta}) \\
& -\frac{1}{2c\sigma^2}\beta^T X^T X\beta\}
\end{aligned} \quad (4)$$

Where, $\hat{\beta} = (X^TX)^{-1}X^TY$

$$
\begin{aligned}
P(\sigma^2/.) &\propto \sigma^{-2(\frac{\nu+n+q}{2}+1)} \\
&\quad exp\{-\frac{1}{2\sigma^2}\left((Y-X\beta)^T(Y-X\beta)+\frac{\beta^TX^TX\beta}{c}+\nu\lambda\right)\} \\
\sigma^2|. &\sim IG\left(\frac{\nu+n+q}{2},\frac{1}{2}(Y-X\beta)^T(Y-X\beta)+\frac{\beta^TX^TX\beta}{2c}+\frac{\nu\lambda}{2}\right) \quad (5)
\end{aligned}
$$

$$
\begin{aligned}
P(c/.) &\propto c^{-(q+1)/2-1} \\
&\quad exp\{-\frac{1}{2c}\left(n+\frac{\beta^TX^TX\beta}{\sigma^2}\right)\} \\
c|. &\sim IG\left(\frac{q+1}{2},\frac{\beta^TX^TX\beta}{2\sigma^2}+n/2\right) \quad (6)
\end{aligned}
$$

$$
\begin{aligned}
P(\beta,|.) &\propto exp\{-\frac{1}{2\sigma^2}(\beta-\hat{\beta})^TX^TX(\beta-\hat{\beta})-\frac{1}{2c\sigma^2}\beta^TX^TX\beta\} \\
\beta|. &\sim N\left(\frac{c}{c+1}\hat{\beta},\frac{c\sigma^2}{c+1}(X^TX)^{-1}\right) \quad (7)
\end{aligned}
$$

1.4 Convergence of sampler

Convergence of the MCMC sampler was assessed by running 10 independent chains and diagnostics of MCMC chain was performed using R-package “coda”. In general, we found that the Markov chains converge within 5000 iterations of the sampler.

1.5 Initialization

We use univariate-eQTL to initialize different parameter of the eQTeL model.

Supplementary Note 2: Further investigation into the reasons for eQ-TeL’s performance gain

In this paper, we have chosen to compare performance of eQTeL against eqtnminer since it is the only method that mostly explicitly incorporated epigenomic data in eQTL as opposed to traditional eQTL approaches. First eqtnminer estimates Bayesian factor (likelihood of association) of each SNP, assuming at most one SNP per gene to be causal; this assumption can be limiting, because it cannot identify combination of SNPs that jointly explain the expression variance. It then estimates posterior probability of each SNP to be causal regulator by modeling prior probability as a function of epigenetic data. However, eqtnminer parameter estimation relies on maximizing a likelihood function, which is prone

to get stuck in local maxima due to correlation among different types of epigenetic data (demonstrated in supplementary note 6 and Fig S9). Further, they do not explicitly model relative weights of genetic and epigenetic factors in determining causality of SNPs. Another approach by Lee et al. [2], does not have the limiting assumption of single causal SNP per gene but it does not incorporate epigenomic data, making comparison infeasible. Recently, Lappalainen et al. [6] uses Matrix-eQTL (essentially a univariate eQTL method) to find associated SNPs, and estimates the proportion of causal SNPs by comparing their epigenomic profiles with that of the most associated SNP per gene as a gold standard (which is a strong assumption). Since they do not explicitly identify causal SNPs amongst associated SNP (the only estimate proportion of causal SNPs), this method is not directly comparable with our method.

To assess performance of eQTeL, we also chose LASSO as a representative of multivariate regression eQTL approaches, because of its good performance and scalability to larger datasets. Other approaches to date [7, 8, 9] that identify causal variants in GWAS, but not in eQTL studies and therefore are not directly comparable.

eQTeLs performance gain is potentially due to two main factors (i) integration of epigenetic data, (ii) allowing multiple causal variants per gene (cite http://www.ncbi.nlm.nih.gov/pubmed/25104515). In quantifying the relative contribution of each of these factors, we note that the mean correlation between actual and predicted eQTeL-predicted gene expression, when a single causal SNP per gene is allowed, is 0.154. This correlation improved substantially to 0.289 when 5 causal SNPs per gene are allowed in eQTeL (Fig S8). However, in the absence of epigenomic data, i.e., when using standard LASSO, we do not see any such performance gain, and in general, the performance is substantially worse than that for eQTeL. This strongly suggests that allowing multiple SNP per gene is useful in identifying regulatory SNP specifically when functional information is used.

Another advantage of eQTeL is that it models heterogeneity in epigenetic signatures of expression regulators. eQTeL is a hierarchical Bayesian model as opposed to empirical Bayes model. Unlike empirical Bayes, hyper-parameters of model are drawn from unparameterized distributions. For this reason in eQTeL all parameters are estimated using MCMC sampling and EM approximation was not required. Empirical prior models [10, 2, 11, 12] assumes a single signature for all regulators and therefore cannot account heterogeneity in the type of regulators of different genes. The eQTeL accommodates such heterogeneity because it allows variation in parameter combinations.

Supplementary Note 3: Other methods for comparison

3.1 Eqtnminer

The software tool related to Gaffney et. al. was downloaded from http://eqtnminer.sourceforge.net. For each of the comparative analysis, the ini-

tial set of SNPs per gene was kept same for both eqtnminer and eQTeL for fair comparison. We obtained Bayesian factor for each SNPs using eqtnminer. The parameters to calculate epigenetic prior were estimated using maximizing equation (9) of Veyrieras et. al. The parameters were initialized as recommended by Veyrieras et. al.

To generate Fig 2, we controlled for total number of SNPs selected by eQTeL and eqtnminer. To do so, we sorted SNPs based on eqtnminer prior probability and selected top 2428 SNPs. As Gaffney et. al. recommend the eqtnminer for single SNP per gene, we compared the performance of eqtnminer in main manuscript (Fig 5, 6 and 8) using single SNP per gene for footprint enrichment, allele-specificity and ChiA-PET enrichment analyses. We repeated that analysis by controlling for number of SNPs per gene between eQTeL and eqtnminer; the eQTeL still outperform eqtnminer in that case. To generate Fig S8, for each gene we selected N (= 1,2, 3 and 5) top SNP(s) based on eqtnminer posterior probability.

3.2 LASSO

R-package GLMNET was used for L1 regulaizer multivariate regression (LASSO). LASSO estimates effect size (regression coefficient), for the SNP included in the model. We used 10-fold cross validation to estimate the hyper-parameter (lambda, regularization parameter). For each of the comparative analysis, the initial set of SNPs per gene was kept the same for both LASSO and eQTeL for fair comparison.

To generate Fig 2, we controlled for total number of SNPs selected by eQTeL and LASSO. To do so, we sorted SNPs based on absolute value of effect size estimated by LASSO-selected top 2428 SNPs. To generate Fig S8, for each gene we selected N (=1,2,3 and 5) top SNP(s) based on absolute value of estimated effect size estimated by LASSO.

3.3 Matrix-eQTL /univariate-eQTL (Lappalainen et. al.)

We used R package matrix-eQTL (http://www.bios.unc.edu/research/genomic_software/Matrix_eQTL/), to perform univariate-eQTL as recommended by Lappalainen et. al.

3.4 Epigenetic-only model

In simulation study, $\boldsymbol{\alpha}$ parameters were learned, in supervised manner, by using enhancers as training example. Bayesian logistic regression [5] was used to learn $\boldsymbol{\alpha}$. Based on learned $\boldsymbol{\alpha}$, SNPs were sorted based on their regulatory potential.

3.5 Known-epigenetic-prior-eQTeL

Known-epigenetic-prior-eQTeL, is a version of eQTeL (for simulation study only) where instead of estimating $\boldsymbol{\alpha}$, the $\boldsymbol{\alpha}$ used to generate regulatory po-

tential for simulation study was used. Thus it is a theoretically best model for eQTeL.

3.6 Variable selection method

Variable selection model was implemented by modifying eQTeL model as follows: (a) informative prior was changed to uninformative priors. (b) hierarchical sampling SNP (based on LD block) was switched off; each SNP were processed sequentially, similar to Liang et. al. [13].

3.7 Lirnet

Lirnet was downloaded from (`http://homes.cs.washington.edu/~suinlee/lirnet/`). Because of computational limitation of lirnet (it takes 13 days of CPU processing in a 64 core machine to process 200 genes), this analysis was limited to 200 random genes. Hyper-parameter of the model was set by cross-validation as recommended in Lee et. al. [2]. For comparing the performance of Lirnet with eQTeL we ran eQTeL with same set of 200 genes.

Figure 13 demonstrates that eQTeL outperforms Lirnet in terms of explained variance and prediction accuracy (we controlled for number of SNPs selected by each methods). Figure 8 also demonstrates that higher fraction of of SNPs detected by eQTeL overlaps with footprints, suggesting eeSNPs are more likely to be functional compared to SNPs detected by Lirnet.

Supplementary Note 4: Eqtnminer subset selection

We used 95 dimensional epigenetic and interaction features, (Fig 2) to learn interacting-regulatory potential by eQTeL. Many of the features have very high correlation between them. When the 95 dimensional features were used for learning prior in eqtnminer, the alpha parameters (feature importance) were not learned accurately. This is most probably due extreme correlation between different input features that might cause the maximization function to stuck in a local maximum. To analyze this further, we used 8 features of the 95 dimensional features, which were given high feature importance by eQTeL and does not have extreme correlation. The performance improved substantially, although eQTeL performed better compared to eqtnminer(Supplementary Fig. 9).

Supplementary Note 5: Multiple hypothesis correction/sparsity con-strains

Here we demonstrate that eQTeL model can detect causal expression-regulatory SNP even if they have small effect size by analyzing sparsity constraints by

association methods on the simulated dataset. Normally, due to multiple-hypothesis test correction (equivalent to sparsity constraint in Bayesian models), expression-regulators with small effect on expression are missed. Fig. 6. shows effect-size distribution of identified causal SNPs by univariate-eQTL and eQTeL when the same number of SNPs is selected by each methods. Univariate-eQTL cannot identify causal SNPs with low effect-size because of severe multiple-hypothesis correction. eQTeL, however, detects causal SNPs with small effect-size. Although the recall-rate decreases with the effect size for eQTeL it can more effectively retrieve causal SNP with small effect size, particularly those with relatively high interacting-regulator potential. Since there are fewer SNPs which are within an interacting-regulator, selection of expression regulators among those SNPs can be made under a relatively less severe sparsity constraint (or equivalently, multiple hypothesis correction). This is evident from from Fig. 6. Moreover, recall rate of eQTeL is relatively higher for top 50% causal SNPs with stronger interacting-regulatory potential (eQTeL-high) than for the bottom 50%. This suggests that eQTeL applies a relatively lower sparsity constraint on interacting-regulators.

Supplementary Note 6: Explained variance and expression predictability

Different methods are known have biases in estimating effect size β. For instance, LASSO is know to over-shrink the parameters, therefore it is recommended that first LASSO be used for feature selection and then β be estimated independently for selected features[14]. To remove such biases and compare performance of different methods in an unbiased manner, each methods were used for regulatory SNPs identification only and β was independently estimated using cross-validation training set as follows.

For each method, explained variance and expression predictability was estimated using k fold cross-validation. Samples were randomly partitioned into k subsamples. $k-1$ of subsamples were used for estimating β for selected SNPs as $\hat{\beta}_{train} = (X^T X)^{-1} X^T Y$, while retaining one subsample for validation. In the validation subsample expression was predicted as $\hat{Y}_{test} = X^T \hat{\beta}_{train}$. Expression predictability was defined as Pearson correlation between Y_{test} and $\hat{Y}_{test}$. Explained variance was calculated as $1 - \frac{var(Y_{test} - \hat{Y}_{test})}{var(Y_{test})}$. This process is repeated k times, using each k subsamples for validation exactly once. The mean and standard deviation of explained variance of expression predictability and explained variance was calculated for k test subsamples.

Supplementary Note 7: Scalability and computation

eQTeL uses shared memory multiprocessing to process genes in parallel. This makes it feasible to run Gibbs sampler to process thousands of genes with million of putative SNPs. In order to calculate Bayesian factor of SNP, we use fast

Choleksy-update algorithm described in Dongarra et al. (Ch 10. [15]). Further, while calculating feature importance $\boldsymbol{\alpha}$ at each Gibbs iteration we randomly sample subset of interacting-regulator and non-regulators to: a) speed up the eQTeL model and b) avoid over-fitting while estimating $\boldsymbol{\alpha}$.

The software GOAL that implements the eQTeL model uses the multiple cores to speed up the process. In addition, we use several efficient algorithms from LAPACK to efficiently update the Choleskythe most computation intensive part of eQTeL. GOAL can efficiently handle million of SNPs for thousand of genes because it process each genes in parallel in a separate thread. In addition, the epigenetic importance can be estimated using subset of genes; and given the importance estimated each of genes could be processed independently.

Supplementary References

[1] Lonsdale, J. *et al.* The genotype-tissue expression (gtex) project. *Nature genetics* **45**, 580–585 (2013).

[2] Lee, S.-I. *et al.* Learning a prior on regulatory potential from eQTL data. *PLoS genetics* **5**, e1000358 (2009). URL http://www.pubmedcentral.nih.gov/articlerender.fcgi?artid=2627940&tool=pmcentrez&rendertype=abstract.

[3] Smith, A. F. & Roberts, G. O. Bayesian computation via the gibbs sampler and related markov chain monte carlo methods. *Journal of the Royal Statistical Society. Series B (Methodological)* 3–23 (1993).

[4] Purcell, S. *et al.* Plink: a tool set for whole-genome association and population-based linkage analyses. *The American Journal of Human Genetics* **81**, 559–575 (2007).

[5] Polson, N., Scott, J. & Windle, J. Bayesian inference for logistic models using Polya-Gamma latent variables. *Journal of the American Statistical ...* 1–42 (2013). URL http://www.tandfonline.com/doi/abs/10.1080/01621459.2013.829001. arXiv:1205.0310v3.

[6] Lappalainen, T. *et al.* Transcriptome and genome sequencing uncovers functional variation in humans. *Nature* (2013).

[7] Valdar, W., Sabourin, J., Nobel, A. & Holmes, C. C. Reprioritizing genetic associations in hit regions using lasso-based resample model averaging. *Genetic epidemiology* **36**, 451–462 (2012).

[8] Zuber, V., Silva, A. P. D. & Strimmer, K. A novel algorithm for simultaneous snp selection in high-dimensional genome-wide association studies. *BMC bioinformatics* **13**, 284 (2012).

[9] Kircher, M. *et al.* A general framework for estimating the relative pathogenicity of human genetic variants. *Nature genetics* **46**, 310–315 (2014).

[10] Gaffney, D. J. *et al.* Dissecting the regulatory architecture of gene expression qtls. *Genome Biol* **13**, R7 (2012).

[11] Kichaev, G. *et al.* Integrating functional data to prioritize causal variants in statistical fine-mapping studies. *PLoS genetics* **10**, e1004722 (2014).

[12] Pickrell, J. K. Joint analysis of functional genomic data and genome-wide association studies of 18 human traits. *American Journal of Human Genetics* **94**, 559–573 (2014). URL http://dx.doi.org/10.1016/j.ajhg.2014.03.004. arXiv:1311.4843v3.

[13] Liang, F., Paulo, R., Molina, G., Clyde, M. a. & Berger, J. O. Mixtures of g Priors for Bayesian Variable Selection. *Journal of the American Statistical Association* **103**, 410–423 (2008). URL http://www.tandfonline.com/doi/abs/10.1198/016214507000001337.

[14] Hastie, T. *et al.* *The elements of statistical learning*, vol. 2 (Springer, 2009).

[15] Dongarra, J. J., Bunch, J. R., Moler, C. B. & Stewart, G. W. *LINPACK users' guide*, vol. 8 (Siam, 1979).
